# Transgenic fluorescent zebrafish lines that have revolutionized biomedical research

**DOI:** 10.1186/s42826-021-00103-2

**Published:** 2021-09-08

**Authors:** Chong Pyo Choe, Seok-Yong Choi, Yun Kee, Min Jung Kim, Seok-Hyung Kim, Yoonsung Lee, Hae-Chul Park, Hyunju Ro

**Affiliations:** 1 Division of Life Science, Gyeongsang National University, Jinju, 52828 Republic of Korea; 2 Division of Applied Life Science, Plant Molecular Biology and Biotechnology Research Center, Gyeongsang National University, Jinju, 52828 Republic of Korea; 3 Department of Biomedical Sciences, Chonnam National University Medical School, Hwasun, 58128 Republic of Korea; 4 Division of Biomedical Convergence, College of Biomedical Science, Kangwon National University, Chuncheon, 24341 Republic of Korea; 5 Department of Biological Sciences, Sookmyung Women’s University, Seoul, 04310 Republic of Korea; 6 Department of Marine Life Sciences and Fish Vaccine Research Center, Jeju National University, Jeju, 63243 Republic of Korea; 7 Center for Genomic Integrity, Institute for Basic Science (IBS), Ulsan, 44919 Republic of Korea; 8 Department of Biomedical Sciences, College of Medicine, Korea University, Ansan, 15355 Republic of Korea; 9 Department of Biological Sciences, College of Bioscience and Biotechnology, Chungnam National University, Daejeon, 34134 Republic of Korea

**Keywords:** Zebrafish, Transgenic, Fluorescent, Signal, Skeletal, Hematopoietic, Nervous, Urogenital, Digestive, Organelle

## Abstract

Since its debut in the biomedical research fields in 1981, zebrafish have been used as a vertebrate model organism in more than 40,000 biomedical research studies. Especially useful are zebrafish lines expressing fluorescent proteins in a molecule, intracellular organelle, cell or tissue specific manner because they allow the visualization and tracking of molecules, intracellular organelles, cells or tissues of interest in real time and in vivo. In this review, we summarize representative transgenic fluorescent zebrafish lines that have revolutionized biomedical research on signal transduction, the craniofacial skeletal system, the hematopoietic system, the nervous system, the urogenital system, the digestive system and intracellular organelles.

## Background

**Fig. 1:**
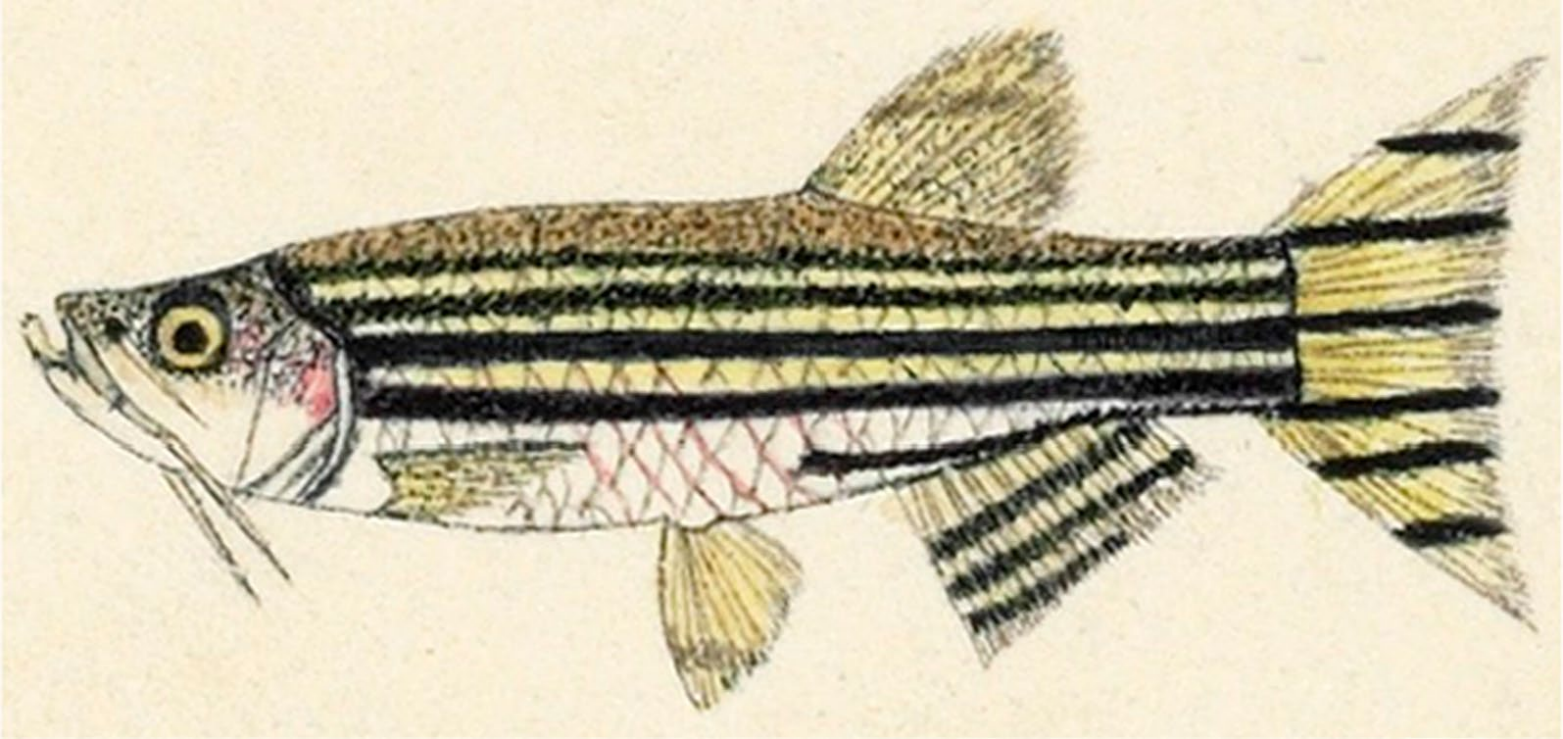
Illustration of zebrafish drawn by Francis Hamilton. Credit: ©The Trustees of the Natural History Museum, London, United Kingdom

Zebrafish (*Danio rerio* or *Brachydanio rerio*) are freshwater fish native to South Asia and were first described scientifically in 1822 by the Scottish physician Francis Hamilton in the monograph entitled *An account of the fishes found in the river Ganges and its branches*, an ichthyological masterpiece [1]. In this monograph, he referred to the zebrafish as “this beautiful fish I found in the Kosi river, where it grows to about two inches in length (Fig. 1).” The genus name ‘*Danio*’ came from the Bengali word ‘Dhani,’ which means rice or rice paddy. One and a half century later, zebrafish made its debut in the biomedical research fields with a George Streisinger’s milestone paper entitled *Production of clones of homozygous diploid zebra fish* (Brachydanio rerio) [2]. In 1988, the Westerfield group first reported that foreign DNA fragments (5.2-kb linearized plasmid) could be integrated into the zebrafish genome and transmitted through the germline [3]. In 1997, the Lin group reported the creation of a transgenic zebrafish line expressing green fluorescent protein (GFP) in a cell-specific manner. They microinjected into one-cell stage zebrafish embryos promoter sequences of *GATA-1*, an erythroid-specific transcription factor, fused to GFP, and confirmed expression of GFP in the *GATA-1*^+^ cells in F1 and F2 zebrafish [4]. Since then, numerous transgenic zebrafish lines expressing fluorescent proteins in a molecule, intracellular organelle, cell or tissue specific manner (referred to as transgenic fluorescent zebrafish lines hereafter) have been generated. As zebrafish embryos are small and transparent, molecules, intracellular organelles, cells or tissues of interest in the transgenic fluorescent zebrafish lines can be easily visualized and tracked in real time and in vivo. As such, they have revolutionized biomedical research on cell biology, developmental biology, genetics, toxicology, human disease pathobiology and drug development. As of April 29, 2021, the Zebrafish Information Network (ZFIN) [5] displays 8960 transgenic zebrafish lines expressing GFP.

In this review, we summarize the representative transgenic fluorescent zebrafish lines that have been extensively used in biomedical research as well as some of their primary applications.

## Main text

### Signal transduction

Early embryonic zebrafish development is tightly regulated by various signaling cascades that should be sequentially activated in restricted domains and time windows [6, 7]. The default state of the fertilized embryos is the ventral fate, determined by the expression of maternal genes related to Transforming growth factor beta (TGF-β) including *radar, bone morphogenetic protein 2 (bmp2), bmp4* and *bmp7* [7–9]. Ventral tissue specification during early embryonic development is abruptly interrupted at the onset of maternal-zygotic transition stage by the expression of various BMP antagonists such as *bozozok, noggin* and *chordin*. The expression of these genes is triggered by the canonical Wnt (Wnt/β-catenin) and Fibroblast growth factor (FGF) signaling, and is restricted to putative organizer regions [7, 10]. Formation of these organizer regions is a prerequisite for the subsequent anterior–posterior (AP) body axis specification. The AP patterning is a complex process triggered by maternal Wnt/β-catenin signaling whose activity is required for the induction of Nodal, another TGF-β related signaling, whereby FGF expression is stimulated in both organizer and blastoderm margin [6, 7]. Ectopically expressed Nodal results in severe embryonic dorsalization as it antagonizes BMP signaling, while the depletion of Nodal disrupts mesendoderm specification, leaving ectoderm unaffected [6, 7]. These signaling pathways also play critical roles in later embryonic development, especially in left–right patterning and in organogenesis.

Various transgenic zebrafish lines have been generated to study the aforementioned signaling pathways as well as the consequences of their hypo- or hyper-activation. To study the role of BMP signaling, Pyati et al. generated a transgenic zebrafish line integrating into its genome a dominant negative BMP receptor fused to GFP under the control of heat shock promoter [*Tg(hsp70l:dnBmpr-GFP)*] (Table 1). With this transgenic line, they were able to delineate the roles of BMP signaling before and after mid-gastrulation by exposing the line to heat shock at different developmental stages [11]. To visualize BMP signaling in live embryos, several transgenic lines have been generated expressing various fluorescent proteins under the control of BMP responsive element adopted from mouse *inhibitor of differentiation-1* enhancer: *Tg(hsp70l:dnBmpr-GFP)*, *Tg(BmpRE-AAVmlp:EGFP)*, *Tg(BRE-AAVmlp:d2GFP)* and *Tg(BRE-AAVmlp:dmKO2)* [12–15]. EGFP indicates enhanced GFP. To track Nodal activity, Dubrelle et al. measured the nuclear accumulation of Smad2, the hallmark of Nodal signaling activation. To accurately quantify the nucleo-cytoplasmic ratio of Smad2, they generated a transgenic zebrafish line expressing GFP-Smad2 ubiquitously, and then observed the graded nuclear accumulation of GFP-Smad2 along the diffused Nodal gradient from vegetal to animal axis during blastula stage [16].

**Table 1:** Transgenic reporter zebrafish lines targeting signal transduction pathways

Construct name	Construct ID	Transgenic name	Transgenic ID	Target	References
*Tg(hsp70l:dnXla.Bmpr1a-GFP)*	ZDB-TGCONSTRCT-070117-43	*Tg(hsp70l:dnBmpr-GFP)*^*w30*^	ZDB-ALT-050503-2	BMP signaling	[11]
*Tg(BMPRE:mRFP)*	ZDB-TGCONSTRCT-110705-4	*Tg(BmpRE:mRFP)*^*cj100*^	ZDB-ALT-110705-4	BMP signaling	[13, 14]
*Tg(BMPRE-AAV.Mlp:EGFP)*	ZDB-TGCONSTRCT-110308-1	*Tg(BmpRE-AAVmlp:EGFP)*^*mw29*^	ZDB-ALT-110308-1	BMP signaling	[15]
*Tg(BRE-AAVmlp:d2GFP)*	ZDB-TGCONSTRCT-110310-1	*Tg(BRE-AAVmlp:d2GFP)*^*mw30*^	ZDB-ALT-110310-1	BMP signaling	[15]
*Tg(BRE-AAVmlp:dmKO2)*	ZDB-TGCONSTRCT-110310-2	*Tg(BRE-AAVmlp:dmKO2)*^*mw40*^	ZDB-ALT-110310-2	BMP signaling	[15]
*Tg(top:GFP)*	ZDB-TGCONSTRCT-070117-137	*Tg(TOP:GFP)*^*w25*^	ZDB-ALT-020621-4	Wnt signaling	[17, 18]
*Tg(7xTCF-Xla.Sia:GFP)*	ZDB-TGCONSTRCT-110113-1	*Tg(7xTCF-Xla.Siam:GFP)*^*ia4*^	ZDB-ALT-110113-1	Wnt signaling	[19]
*Tg(7xTCF-Xla.Sia:NLS-mCherry)*	ZDB-TGCONSTRCT-110113-2	*Tg(7xTCF-Xla.Siam:nlsmCherry)*^*ia5*^	ZDB-ALT-110113-2	Wnt signaling	[19]
*Tg(kdrl:EGFP)*	ZDB-TGCONSTRCT-070117-47	*Tg(kdr:EGFP)*^*s843*^	ZDB-GENO-050916-7	Vascular endothelial cells	[19]
*Tg(Tie2:EGFP)*	ZDB-TGCONSTRCT-070117-80	*Tg(Tie2:EGFP)*^*s849*^	ZDB-ALT-050916-12	Vascular endothelial cells	[19]
*Tg(myl7:EGFP)*	ZDB-TGCONSTRCT-070117-164	*Tg(myl7:EGFP)*^*twu34*^	ZDB-ALT-050809-20	Heart muscle	[19]
*Tg(sox10:mRFP)*	ZDB-TGCONSTRCT-080321-2	*Tg(sox10:mRFP)*^*vu234*^	ZDB-ALT-080321-3	Olfactory bulb, iridophore, glial cells, neural crest and nervous system	[19]
*Tg(hsp70l:dkk1b-GFP)*	ZDB-TGCONSTRCT-070403-1	*Tg(hsp70l:dkk1-GFP)*^*w32*^	ZDB-ALT-070403-1	Dkk1b	[19]
*Tg(hsp70l:wnt8a-GFP)*	ZDB-TGCONSTRCT-070403-3	*Tg(hsp70:wnt8a-GFP)*^*w34*^	ZDB-ALT-070403-3	Wnt8a	[19]
*Tg(fli1:EGFP)*	ZDB-TGCONSTRCT-070117-94	*Tg(fli1:EGFP)*^*y1*^	ZDB-ALT-011017-8	Pharyngeal arch, endothelial cells and skeletogenic precursors	[19, 20, 23]
*Tg(dusp6:d2EGFP)*	ZDB-TGCONSTRCT-071017-3	*Tg(dusp6:d2EGFP)*^*pt6*^	ZDB-ALT-071017-3	FGF signaling	[20]
*Tg(hsp70l:dnfgfr1a-EGFP)*	ZDB-TGCONSTRCT-070117-101	*Tg(hsp70l:dnfgfr1-EGFP)*^*pd1*^	ZDB-ALT-060322-2	FGF signaling	[21, 22]
*Tg(fabp10a:dnfgfr1-EGFP)*	ZDB-TGCONSTRCT-131120-6	*Tg(fabp10a:dnfgfr1-EGFP)*^*zf421*^	ZDB-ALT-131120-24	FGF signaling specifically in liver	[22]

Several lines are available to study Wnt/β-catenin signaling in live embryos. In the *Tg(top:GFP)* transgenic line, the fluorescent reporter is under the control of TOPFlash comprising four consensus TCF/LEF binding elements juxtaposed to a *c-fos* minimal promoter [17, 18]. Also developed are transgenic fluorescent zebrafish lines in which GFP or nuclear mCherry is driven by the enhancer region of minimal promoter of *Xenopus* gene *siamois* with seven tandem repeats of the TCF/LEF binding elements: *Tg(7xTCF-Xla.Siam:GFP)*, *Tg(7xTCF-Xla.Siam:nlsmCherry)*, *Tg(kdr:EGFP)*, *Tg(Tie2:EGFP)*, *Tg(myl7:EGFP)*, *Tg(sox10:mRFP)*, *Tg(hsp70l:dkk1-GFP)*, *Tg(hsp70:wnt8a-GFP)* and *Tg(fli1:EGFP)* [19]. With these reporter lines, Moro et al. identified zebrafish tissues sensitive to canonical Wnt stimuli including the hypothalamus, the gill arches and rakers, the olfactory bulb and the cloacal aperture. In contrast to the canonical Wnt signaling, transgenic zebrafish lines have not been used to study non-canonical Wnt signaling, to the best of our knowledge. Therefore, it would be challenging to explore zebrafish planar cell polarity governed by the non-canonical Wnt signaling in real time and in vivo.

As the endogenous expression of *dual specificity phosphatase 6* (*dusp6*, also called *Mkp3*) is directly controlled by FGF signaling throughout zebrafish development, Molina et al. introduced destabilized EGFP under the control of the isolated *dusp6* promoter. The resulting *Tg*(*dusp6:EGFP*) is highly sensitive to FGF signaling and allows for the visualization of FGF responsive tissues [20]. Moreover, as *Tg*(*dusp6:EGFP*) is easily sensitized by specific FGF signaling chemical antagonists, this reporter embryos are very useful to identify compounds that alter FGF signaling. On the other hand, to suppress FGF signaling in developing embryos, dominant negative FGF receptor (*dn-fgfr1*) was exploited for the transgenesis. Initially, Hsp70 promoter was adopted to generate *Tg(hsp70l:dnfgfr1-EGFP)* zebrafish that can be activated by moderate heat stimuli [21]. Later on, Tsai et al. generated transgenic zebrafish in which *dn-fgfr1* is expressed specifically in the liver using a hepatocyte specific promoter, a *liver fatty acid binding protein* (*lfabp*) promoter. This adult *Tg(fabp10a:dnfgfr1-EGFP)* manifested liver pathology such as hepatic steatosis and cholestasis, suggesting that the maintenance of hepatic FGF signaling is required for lipid homeostasis [22].

### The craniofacial skeletal system

#### Introduction

Formation of the craniofacial skeleton is an intricate process that requires a series of developmental events mediated by cells derived from the neural crest (NC), endoderm, mesoderm, and ectoderm. The craniofacial skeleton is largely derived from cranial neural crest (CNC) cells that populate the pharyngeal arches [24, 25]. From there, CNC cells become skeletogenic precursors called ectomesenchyme and then chondrocytes and osteoblasts that will subsequently form facial cartilages and bones, respectively [24–26]. While the CNC is an important transient cell population for the development of craniofacial skeleton, the pharyngeal endoderm, mesoderm, and ectoderm are required for the CNC to form the craniofacial skeleton. During embryogenesis, the head ectoderm forms a series of infoldings, termed clefts, and the pharyngeal endoderm develops a series of outpocketings, termed pouches [27, 28]. The endodermal pouches, together with the ectodermal clefts, segment the pharyngeal arches and have important signaling functions in the survival and differentiation of CNC cells during craniofacial skeleton development [27, 29]. While the head mesoderm of the pharyngeal arches is primarily of myogenic origin, it also contributes to craniofacial skeleton development by establishing and patterning the pharyngeal endoderm [27, 30]. Thus, to better understand craniofacial skeleton development, essentially required is the unprecedented ability to track and manipulate cells derived from the NC, endoderm, mesoderm and ectoderm in vivo. To this end, several transgenic fluorescent zebrafish lines have been generated (Table 2) and widely used for research on craniofacial development.

**Table 2:** Transgenic reporter zebrafish lines targeting the craniofacial-skeletal system

Construct name	Construct ID	Transgenic name	Transgenic ID	Target	References
*Tg(fli1:EGFP)*	ZDB-TGCONSTRCT-070117-94	*Tg(fli1:EGFP)*^*y1*^	ZDB-ALT-011017-8	Pharyngeal arch, endothelial cells and skeletogenic precursors	[23]
*Tg(sox10:GAL4-VP16-IRES-EGFP)*	ZDB-TGCONSTRCT-120523-1	*Tg(sox10:GAL4-VP16-IRES-EGFP)*^*el159*^	ZDB-ALT-120523-1	NCs and CNS	[34]
*Tg(sp7:EGFP)*	ZDB-TGCONSTRCT-100402-2	*Tg(sp7:EGFP)*^*b1212*^	ZDB-ALT-100402-1	Osteoblasts	[41]
*Tg(Hsa.RUNX2-Mmu.Fos:EGFP)*	ZDB-TGCONSTRCT-120209-6	*Tg(Hsa.RUNX2-Mmu.Fos:EGFP)*^*zf259*^	ZDB-ALT-120209-60	Osteoblasts	[46–49]
*Tg1(Ola.Bglap:EGFP)*	ZDB-TGCONSTRCT-110713-1	*Tg1(Ola.Bglap:EGFP)*^*hu4008*^	ZDB-ALT-110713-1	Late osteoblasts	[36, 42, 46, 47]
*Tg(rr.col2a1a.2:EGFP)*	ZDB-TGCONSTRCT-111205-2	*Tg(rr.col2a1a.2:EGFP)*^*nu13*^	ZDB-ALT-111205-4	Chondrocytes	[54]
*Tg(-1.7col2a1a:EGFP-CAAX)*	ZDB-TGCONSTRCT-111205-1	*Tg(-1.7col2a1a:EGFP-CAAX)*^*nu12*^	ZDB-ALT-111205-1	Chondrocytes	[57]
*Tg(foxp2.A:EGFP)*	ZDB-TGCONSTRCT-100412-4	*Tg(foxp2.A:EGFP)*^*zc42*^	ZDB-ALT-100412-5	Chondrocytes	[60, 61]
*Tg(foxp2.A:EGFP)*	ZDB-TGCONSTRCT-100412-4	*Tg(foxp2.A:EGFP)*^*zc81*^	ZDB-ALT-111214-1	Chondrocytes	[62–64]
*Tg(her5PAC:EGFP)*	ZDB-TGCONSTRCT-070117-155	*Tg(her5PAC:EGFP)*^*ne1939*^	ZDB-ALT-050919-8	Mid-hindbrain, pharyngeal endoderm and pouch	[65]
*Tg(-3.4her5:EGFP)*	ZDB-TGCONSTRCT-070117-9	*Tg(-3.4her5:EGFP)*^*ne1911*^	ZDB-ALT-050919-4	Mid-hindbrain and pharyngeal endoderm	[65]
*Tg(sox17:GFP)*	ZDB-TGCONSTRCT-070117-57	*Tg(sox17:GFP)*^*s870*^	ZDB-ALT-061228-2	Pharyngeal endoderm	[74–77]
*Tg(nkx2.3:GAL4-VP16,myl7:EGFP)*	ZDB-TGCONSTRCT-130610-1	*Tg(nkx2.3:GAL4-VP16,myl7:EGFP)*^*el93*^	ZDB-ALT-130610-2	Pharyngeal pouch	[67]
*Tg(UAS:CFP-Eco.NfsB,myl7:EGFP)*	ZDB-TGCONSTRCT-130610-7	*Tg(UAS:CFP-Eco.NfsB,myl7:EGFP)*^*el53*^	ZDB-ALT-130610-8	Gal4/UAS system	[67]
*Tg(nkx2.3:EGFP-dvl2)*	ZDB-TGCONSTRCT-130610-3	*Tg(nkx2.3:EGFP-dvl2)*^*el303*^	ZDB-ALT-130610-4	Pharyngeal pouch	[67]
*Tg(nkx2.3:alcama-EGFP)*	ZDB-TGCONSTRCT-130610-4	*Tg(nkx2.3:alcama-EGFP)*^*el304*^	ZDB-ALT-130610-5	Pharyngeal pouch	[67]
*Tg(nkx2.5:EGFP)*	ZDB-TGCONSTRCT-120828-5	*Tg(nkx2.5:EGFP)*^*el83*^	ZDB-ALT-120828–8	Cardiogenesis and head mesoderm	[67, 84]
*Tg(nkx2.5:GAL4-VP16,myl7:EGFP)*	ZDB-TGCONSTRCT-130610-2	*Tg(nkx2.5:GAL4-VP16,myl7:EGFP)*^*el74*^	ZDB-ALT-130610-3	Cardiogenesis and head mesoderm	[67]

#### Ectomesenchyme transgenic lines

The Fli1 transcription factor marks endothelial cells as well as CNC-derived skeletogenic precursors [31, 32]. Although the *Tg(fli1:EGFP)* transgenic line was originally generated to visualize endothelial cells [23], it has been widely used to image CNC cells within the pharyngeal arches. Lawson and Weinstein identified fragments containing the 5′ end of the *fli1* gene by screening a P1-derived artificial chromosome (PAC) library, and subcloned a 15-kb PAC DNA fragment into the pGEM3zf plasmid. EGFP was placed immediately upstream of the *fli1* start codon, resulting in the *Tg(fli1:EGFP)* transgenic construct, which was linearized with NotI and then injected into one-cell stage embryos to generate *Tg(fli1:EGFP)* zebrafish. This transgenic zebrafish labels the endothelial cells as well as CNC cells. During craniofacial skeleton development, this line expresses EGFP in post-migratory CNC cells in the pharyngeal arches. To understand the early stage of craniofacial skeleton formation, this line, along with the *Tg(sox10:EGFP)* line that expresses EGFP in NC including the CNC, has been used to visualize the skeletogenic precursors. For example, this line was adopted to study the roles of the variant histone H3.3, BMPs and inhibitor of DNA binding 2a (Id2a) in ectomesenchyme potential of the CNC [33, 34] as well as to correlate defects in pharyngeal pouches with later defects in the condensation of CNC cells to form the craniofacial skeleton [27, 35].

#### Cartilage and bone transgenic lines

In addition to the transgenic reporters marking the CNC and skeletogenic precursors, a number of transgenic lines have been developed to image different cell types within the differentiated skeleton in vivo. These include *Tg(sp7:EGFP)*, *Tg(RUNX2:EGFP)* and *Tg(osteocalcin*:EGFP*)* for osteoblasts, and *Tg(col2a1a:EGFP)* and *Tg(sox9a:EGFP)* for chondrocytes. Sp7 (Osterix) and RUNX2 are expressed in committed osteoblast progenitors, and Osteocalcin (Bone γ-carboxyglutamate protein [Bglap]) in late osteoblasts [36].

Sp7 is a transcription factor that is expressed in osteoblasts but not chondrocytes, making it an excellent marker of osteoblasts [37]. Although a 4.1-kb upstream regulatory region of the medaka *sp7* gene was used to drive mCherry or nuclear GFP expression in zebrafish [38], this transgenic line did not completely recapitulate expression pattern of endogenous zebrafish *sp7* [39, 40]. To overcome this discrepancy, the Kimmel group wished to generate the transgenic *sp7* reporter using a regulatory region of the zebrafish *sp7* [39]. Since the regulatory elements necessary for zebrafish *sp7* expression were not known, they used bacterial artificial chromosome (BAC) mediated transgenesis to drive EGFP expression under the control of the sequences upstream of *sp7*. Using an *E. coli* recombination machinery, they inserted a DNA fragment containing EGFP and a kanamycin resistant cassette into the start codon of the *sp7* coding sequence in a BAC harboring the full *sp7* sequences. The resulting BAC *Tg(sp7:EGFP)* construct was used to generate a transgenic line via BAC transgenesis technology [41]. In this transgenic line, EGFP expression reproduced endogenous *sp7* expression in the otic primordium and skeletal structures, including osteoblasts. In the adult transgenic fish, EGFP was detected in skeletal elements, such as the tail fin bony rays, and regenerating fin rays post-amputation [39]. In vivo imaging of *Tg(sp7:EGFP)* line revealed that a population of *sp7:EGFP*^−^ cells is a key source of repair chondrocytes in jawbone regeneration [42]. In addition, this line contributed to produce epigenome maps, including DNA methylation and chromatin accessibility, in *sp7:EGFP*^+^ osteoblasts from uninjured and regenerating fins [43].

*Tg(RUNX2:EGFP)* is another transgenic line that has been extensively used for in vivo imaging of osteoblasts. The transcription factor Runx2 along with Sp7 is critical to osteoblast differentiation in zebrafish and mammals, rendering it an excellent marker of osteoblasts [4, 44, 45]. The Weidinger group originally generated the *Tg(RUNX2:EGFP)* to study the cellular basis of fin regeneration in adult zebrafish [46]. They utilized a 557-bp multispecies-conserved regulatory element located in the last intron of the human *RUNX2* gene. They cloned it into the Tol2 transposon vector pGWcfosEGFP, containing the *cFos* minimal promoter and EGFP, thereby generating the *Tg(Hsa.RUNX2-Mmu.Fos:EGFP)* construct, with which they created *Tg(Hsa.RUNX2:EGFP)* line using the Tol2 transposon system. This line expresses EGFP in all newly forming bones of the craniofacial and appendicular skeleton from embryo to adult [46], making it suitable to investigate bone development and regeneration. For example, the in vivo imaging of GFP expression or whole-mount in situ hybridization (WISH) against *gfp* RNA in this line demonstrated that mature osteoblasts dedifferentiate to form pre-osteoblasts following fin amputation or bone fracture, and that NF-κB signaling negatively regulates osteoblast dedifferentiation [46–48]. Time-lapse imaging analysis of this line to explore dynamics of skull bone formation showed that Sp7 is required for the maturation and proliferation control of *RUNX2:EGFP*^+^ early osteoblasts [49].

Osteocalcin is a late osteoblast marker. To generate an *osteocalcin* reporter, the Weidinger group first cloned the 3.7-kb promoter sequence of the medaka (*Oryzias latipes*) *osteocalcin* and inserted it into the upstream region of EGFP in pBluescript vector containing I-SceI meganuclease sites flanking the insert [50]. The resulting construct was used to create a osteocalcin reporter line *Tg(osteocalcin*:EGFP*)* using I-SceI mediated transgenesis [46]. This line was used to demonstrate that zebrafish osteoblasts dedifferentiate into osteogenic precursor cells in the fractured bony fin ray and skull [47], and that repair chondrocytes dedifferentiate into osteoblasts during zebrafish jawbone regeneration [42].

A better understanding of craniofacial skeleton development entails transgenic lines that can visualize cartilage development as well. As such, *Tg(col2a1a:EGFP)* and *Tg(sox9a:EGFP)* lines were generated to illuminate chondrocyte formation and distribution during cartilage development. Collagen is the most abundant protein in the extracellular matrix [51, 52] and type II collagen is an important component of cartilage [53]. Zebrafish has two paralogs of *col2a1*, the *col2a1a* and *col2a1b*, of which *col2a1a* is robustly expressed in all craniofacial chondrocytes, making it an excellent marker for this cell type [54]. Dale and Topczewski identified a regulatory element in the *col2a1a* locus, termed R2, located from − 1720 to − 1411 bp of the transcription start site (TSS). Using the Multistep Gateway Recombination system (Invitrogen) and the Tol2kit [55, 56], they fused the R2 element to a minimal promoter containing a carp TATA box and the adenovirus E1b promoter upstream of EGFP, generating the *Tg(rr.col2a1a.2:EGFP)* construct. They also created the *Tg(-1.7col2a1a:EGFP-CAAX)* construct with the entire 1.7-kb fragment upstream of the TSS. Both resulting transgenic lines, *Tg(rr.col2a1a.2:EGFP)* and *Tg(-1.7col2a1a:EGFP-CAAX),* express EGFP in craniofacial cartilage. These lines helped uncover that Wnt9a is required for palatal extension [57], and that Iroquois transcription factor inhibits chondrocyte maturation at the hyoid joint [58]. The entire 1.7-kb upstream sequence was used to drive guanine nucleotide exchange factor subunit RIC1 and its downstream targets in chondrocytes, which revealed the underlying mechanism of RIC1-linked Mendelian syndrome [59].

The transgenic zebrafish line *sox9a*^*zc81Tg*^, commonly known as *Tg(sox9a:EGFP)*, has been also widely used for in vivo imaging of craniofacial chondrocytes. Interestingly, the *Tg(sox9a:EGFP)* line was generated inadvertently. To generate a transgenic reporter visualizing the central nervous system (CNS) during development, the Dorsky group isolated a 9.7-kb DNA fragment from the *foxP2* locus, and used it to build the *Tg(foxp2.A:EGFP)* construct via Gateway technology [60]. They then microinjected the resulting constructs into zebrafish embryos at one-cell stage, and raised the embryos to adulthood. While screening F1 embryos from founder zebrafish, they noted one founder whose embryos expressed EGFP in the pharyngeal arches where endogenous *foxp2* is not expressed. This finding suggested that in the genome of this founder, EGFP expression was driven by a nearby promoter rather than by a *foxP2* promoter per se. Indeed, further investigation revealed that the *Tg(foxp2.A:EGFP)* insertion locus in the genome was located approximately 120-kb upstream of the *sox9a* TSS [61]. As a result, F1 zebrafish from the founder was termed *Tg(sox9a:EGFP)* line instead of *Tg(foxp2.A:EGFP)* line. About 55 h post-fertilization (hpf) at which *Tg(fli1:EGFP)* expression in the CNC-derived ectomesenchyme is abolished in the pharyngeal arches, the *Tg(sox9a:EGFP)* line expresses EGFP in early cartilage rudiments developing in these arches [61]. This line was used to uncover the role of *sox9a*-expressing cells in musculoskeletal integration in the zebrafish jaw [62]. Time-lapse imaging of craniofacial skeleton development in this line revealed that the opercle and the adjacent ceratohyal cartilage migrate in a coordinated manner [63], and that gaps in *sox9a:EGFP*^+^ cartilage exhibit hallmarks of joint identity during craniofacial skeleton development [64].

#### Pharyngeal endoderm transgenic lines

Absence of the endoderm or the pharyngeal endoderm derived pharyngeal pouches leads to severe defects in the craniofacial skeleton of zebrafish, indicating that these tissues are critical to the craniofacial skeleton development [30]. To better understand how the pharyngeal endoderm and pouches regulate craniofacial skeleton development, transgenic reporter lines were needed to visualize these tissues during craniofacial development. The first transgenic reporter illuminating pharyngeal endoderm formation was the *Tg(her5PAC:EGFP)* transgenic line [65], although this line was originally generated to investigate the development of midbrain-hindbrain (MH) domain of the vertebrate embryonic neural tube in vivo. For this purpose, Tallafuss and Bally-Cuif isolated a PAC containing the complete set of regulatory elements of *her5*, the first reported gene expressed in the MH domain, and inserted EGFP within exon 2 of the *her5* genomic region via ET-mediated homologous recombination [66]. The resulting construct was used to establish the *Tg(her5PAC:EGFP)* line via the PAC-mediated transgenesis [65]. In this line, EGFP expression reproduces endogenous *her5* expression in the MH domain and the pharyngeal endoderm, making this line the first transgenic reporter of the pharyngeal endoderm. They also generated the *Tg(-3.4her5:EGFP)* line harboring EGFP driven by 3.4-kb fragment upstream of the *her5* TSS, which mirrors the EGFP expression pattern in the *Tg(her5PAC:EGFP)* line almost completely [65]. Since then, both lines have been extensively used. For example, time-lapse imaging analysis in the *Tg(-3.4her5:EGFP)* line revealed that the pharyngeal pouches are formed by the branching morphogenesis and that Wnt11r and Wnt4a are required for this morphogenesis [67]. This line was also employed to correlate defects in the pharyngeal endoderm with later defects in the craniofacial cartilage development [68–70].

Together with the *Tg(her5:EGFP)* line, the *Tg(sox17:GFP)* line has been used to illuminate development of the endoderm, including the pharyngeal endoderm. In zebrafish and mammalian embryogenesis, a transcription factor Sox17 is the key regulator of the specification and morphogenesis of endodermal epithelia, making it an excellent pan-endoderm marker [71–73]. Chung and Stainier originally generated the *Tg(sox17:GFP)* line to identify progenitors of endodermal organs such as the liver, pancreas and intestine [74]. They first isolated the 4.2-kb upstream regulatory region from the pEGFP1 *sox17* promoter plasmid constructed by Yutaka Kikuchi and cloned it into a Tol2 GFP transposon vector, thereby building the *Tg(sox17:GFP)* construct, with which they created the *Tg(sox17:GFP)* line using the Tol2 transposon system. In this line, GFP is expressed in almost all endodermal cells, including the endodermal cells at 50% epiboly, the progenitors of the liver, pancreas and intestine, the condensing foregut endodermal cells, and the pharyngeal endodermal cells [74–77]. Various studies on the endoderm and craniofacial skeleton development have benefited from the *Tg(sox17:GFP)* line. For example, in vivo imaging of *sox17:GFP*^+^ pharyngeal endoderm correlated defects in the morphogenesis of pharyngeal endoderm with later defects in craniofacial cartilage morphology [76, 78, 79]. In addition, this line has been used to understand the developmental mechanisms underlying formation of the endodermal organs such as the liver, pancreas, and intestine [74, 80, 81].

During craniofacial development, a population of cells in the pharyngeal endoderm forms a series of pouches that governs the development of pharyngeal arches and craniofacial skeleton [27, 30]. To better understand the role for this specific population of cells in the development of craniofacial skeleton, transgenic lines expressing reporter genes specifically in the pouches were needed. Zebrafish *nkx2.3* is specifically expressed in the developing pouches, but not in the pharyngeal endoderm, making it an excellent marker of the pouches [82]. To illuminate the morphogenesis of pouches, the Crump group created a *Tg(nkx2.3:Gal4-VP16)* line that can drive expression of any gene governed by the UAS promoter specifically in the pouches [67]. They isolated a 5.5-kb regulatory region upstream of the *nkx2.3* locus and made the *Tg(nkx2.3:GAL4-VP16,myl7:EGFP)* construct using the Gateway technology, with which they developed the *Tg(nkx2.3:Gal4-VP16,myl7:EGFP)* line using the Tol2 transposon system [67]. The *Tg(UAS:CFP-Eco.NfsB,myl7:EGFP)* line expresses cyan fluorescent protein (CFP) fused to nitroreductase (NTR), an *E. coli* enzyme that converts the prodrug metronidazole (MTZ) to cytotoxin, specifically in the pharyngeal pouches throughout pouch morphogenesis in a pattern consistent with endogenous *nkx2.3* expression [67, 82]. The combination of *Tg(nkx2.3:Gal4-VP16,myl7:EGFP)* line and *Tg(UAS:CFP-Eco.NfsB,myl7:EGFP)* line revealed the pouch-specific requirements for effectors of Wnt and Dvl signaling during pouch morphogenesis. In addition, a number of transgenic constructs has been generated with the 5.5-kb upstream regulatory region of *nkx2.3*, including the *Tg(nkx2.3:EGFP-dvl2)* and *Tg(nkx2.3:alcama-EGFP)*, time-lapse imaging of which showed correlation between the rearrangement of pouch cells and the dynamic subcellular localization of Dishevelled (Dvl) and Alcama during pouch formation [67].

#### Pharyngeal mesoderm transgenic lines

The head mesoderm has been reported as an important source of multiple signaling necessary for pouch formation associated with the craniofacial skeleton development [27, 30]. However, the absence of transgenic reporters that can mark and manipulate the head mesoderm in live animals, has hindered the progress in understanding the cellular and molecular mechanism by which the head mesoderm controls the development of the pharyngeal endoderm and craniofacial skeleton. In zebrafish, *nkx2.5* is the earliest heart field marker, and expressed farther posteriorly to the mesoderm adjacent to the pharyngeal arches and pouches during craniofacial skeleton development [83]. Thus, a transgenic *nkx2.5* reporter was expected to label the head mesoderm. The Crump group isolated a 6-kb upstream regulatory region from the *nkx2.5* locus and established the *Tg(nkx2.5:EGFP)* construct using the Gateway technology [67], with which they created the *Tg(nkx2.5:EGFP)* line with the Tol2 transposon system [67, 84]. This line faithfully reproduced endogenous *nkx2.5* expression in the second heart field during cardiogenesis and in the head mesoderm during craniofacial skeleton development [67, 84]. The *Tg(nkx2.5:EGFP)* line has been used in many studies. For example, time-lapse imaging of pharyngeal pouch formation revealed unexpected dynamic interactions between the head mesoderm and the pharyngeal endoderm during craniofacial skeleton development, along with the roles of Wnt11r and FGF8a in the mesoderm-endoderm interaction [67, 85]. In addition, a number of transgenic constructs, including the *Tg(nkx2.5:GAL4-VP16,myl7:EGFP)*, has been generated with the 6-kb upstream regulatory region of *nkx2.5* [67]. In conjunction with the *Tg(UAS:CFP-NTR)*, the *Tg(nkx2.5:Gal4-VP16,myl7:EGFP)* line uncovered the requirement for *nkx2.5:CFP-NTR*^+^ mesoderm in the development of pouches and craniofacial skeleton [67].

### The hematopoietic system

#### Introduction

The hematopoietic genes and regulatory networks as well as all mature blood lineages and maturation intermediates in zebrafish are evolutionarily conserved [86]. As in mammals, zebrafish hematopoiesis initiates with sequential primitive and definitive waves of blood cells [87]. First, the primitive waves of hematopoiesis produce erythrocytes and macrophages through unipotent precursors from the embryonic mesoderm. Afterwards, the definitive hematopoietic stem cells (HSCs) capable of generating all mature blood cell types arise from the hemogenic endothelium [88]. The transgenic reporter lines labelling each and every type of blood lineages have been introduced over the past two decades, allowing for real time imaging in live zebrafish and purification of blood cells of interest through fluorescence activated cell sorting (FACS) [89]. Here, we summarize the representative zebrafish transgenic reporter lines labeling distinct blood populations (Table 3), which have been used for characterizing various hematopoietic events.

**Table 3:** Transgenic reporter zebrafish lines targeting the hematopoietic system

Construct name	Construct ID	Transgenic name	Transgenic ID	Target	References
*Tg(gata1a:GFP)*	ZDB-TGCONSTRCT-070117-153	*Tg(gata1a:GFP)*^*la781*^	ZDB-ALT-051018-2	Red blood cells	[92]
*Tg(mpx:GFP)*	ZDB-TGCONSTRCT-070118-1	*Tg(mpx:GFP)*^*uwm1*^	ZDB-ALT-070502-1	Neutrophils	[97]
*Tg(mpeg1:EGFP)*	ZDB-TGCONSTRCT-120117-1	*Tg(mpeg1:EGFP)*^*gl22*^	ZDB-ALT-120117-1	Macrophages	[98]
*Tg(mpeg1:mCherry)*	ZDB-TGCONSTRCT-120117-2	*Tg(mpeg1:mCherry)*^*gl23*^	ZDB-ALT-120117-2	Macrophages	[98]
*Tg(-6.0itga2b:EGFP)*	ZDB-TGCONSTRCT-070117-128	*Tg(-6.0itga2b:EGFP)*^*la2*^	ZDB-ALT-051223-4	HSCs	[86]
*TgPAC(myb:2xmyb-EGFP)*	ZDB-TGCONSTRCT-071017-1	*TgPAC(myb:2xmyb-EGFP)*^*zf169*^	ZDB-ALT-071017-1	HSCs	[112]
*Tg(lmo2:DsRed)*	ZDB-TGCONSTRCT-071017-2	*Tg(lmo2:DsRed)*^*zf73*^	ZDB-ALT-080122-9	Endothelium	[112]
*Tg(Mmu.Runx1:EGFP)*	ZDB-TGCONSTRCT-150512-1	*Tg(Mmu.Runx1:EGFP)*^*cz2009*^	ZDB-ALT-150512-1	HSCs	[110]
*Tg(Mmu.Runx1:NLS-mCherry)*	ZDB-TGCONSTRCT-150512-2	*Tg(Mmu.Runx1:NLS-mCherry)*^*cz2010*^	ZDB-ALT-150512-2	HSCs	[110]
*Tg(kdrl:Cre)*	ZDB-TGCONSTRCT-100419-2	*Tg(kdrl:Cre)*^*s898*^	ZDB-ALT-100419-3	Endothelium-specific Cre line	[88]
*Tg(actb2:LOXP-STOP-LOXP-DsRedEx)*	ZDB-TGCONSTRCT-100301-1	*Tg(actb2:LOXP-STOP-LOXP-DsRedEx)*^*sd5*^	ZDB-ALT-100301-1	Cre-mediated genetic switch line	[88]
*TgBAC(gata2b:KALTA4)*	ZDB-TGCONSTRCT-150602-8	*TgBAC(gata2b:KALTA4)*^*sd32*^	ZDB-ALT-150602-13	Hemogenic endothelium-specific Gal4 line	[125]
*Tg(4xUAS:GFP)*	ZDB-TGCONSTRCT-100809-1	*Tg(4xUAS:GFP)*^*hzm3*^	ZDB-ALT-100809-1	Gal4/UAS system	[126, 127]
*Tg(UAS:Cre,cryaa:Venus)*	ZDB-TGCONSTRCT-150602-10	*Tg(UAS:Cre,cryaa:Venus)*^*zd17*^	ZDB-ALT-150602-15	Gal4/UAS system	[125]
*Tg(rag1:GFP)*	ZDB-TGCONSTRCT-070117-55	*Tg(rag1:GFP)*^*la5*^	ZDB-ALT-051001-2	Lymphocytes	[133]
*Tg(rag2:GFP)*	ZDB-TGCONSTRCT-070117-56	*Tg(rag2:GFP)*^*la6*^	ZDB-ALT-051001-4	Lymphocytes	[132]

#### Erythroid transgenic lines

During early embryogenesis, red blood cells (RBCs) arise from the intermediate cell mass (ICM), which is derived from the posterior lateral mesoderm (PLM) [90, 91]. To label RBCs, the Lin group at Medical College of Georgia generated a *Tg*(*gata1a:GFP)* zebrafish by injecting the linearized construct G1-GM2 comprising the zebrafish GATA-1 promoter and GFP (GM2) into embryos [92]. The GATA-1 promoter construct is a 5.6-kb DNA fragment from a GATA-1 genomic fragment containing the 5′ upstream region of the GATA-1 TSS. This line has been used to study embryonic erythropoiesis [92], thrombocyte development [93], hematopoietic transplantation [86], leukemia [94] and real-time cardiac pumping dynamics [95].

#### Myeloid transgenic lines

Myeloid cell populations including macrophages and neutrophils originate from anterior lateral mesoderm [96]. To uncover the molecular and cellular mechanisms regulating neutrophil‐mediated inflammation in vivo, the Huttenlocher group isolated a PAC containing the zebrafish *zMPO* promoter sequences and subcloned an 8‐kb fragment of the *zMPO* 5′‐untranslated region (UTR) into the 5′ region of GFP (*zMPO:GFP*), producing the transgenic line *Tg(mpx:GFP)* where neutrophils express GFP under the control of myeloperoxidase promoter [97]. The Lieschke group developed a macrophage-specific reporter line to track macrophages and their interactions with neutrophils in real time and in vivo [98]. The promoter of a macrophage-specific marker *mpeg1* was utilized to generate the line. The proximal 1.86-kb DNA fragment of *mpeg1* 5′-UTR was subcloned into the 5′ region of either EGFP or mCherry, and Tol2-mediated transgenesis was performed to generate *Tg(mpeg1:EGFP)* or *Tg(mpeg1:mCherry)* that expresses EGFP or mCherry in the macrophages, respectively [98]*. Tg(mpx:GFP)*, *Tg(mpeg1:EGFP)* and *Tg(mpeg1:mCherry)* lines can address behavior and development of myelopoids such as phagocytosis of senescent neutrophils [98]. These lines have been used to take real-time and in vivo images of responses against inflammation [97], wound detection [99], neutrophil motility [100], the role of eosinophils in immunity [101], tissue regeneration [102, 103], cardiac wound healing[104], metabolic diseases [105], microglia development [106], axon degeneration [107] and HSC development [108].

#### HSC transgenic lines

The first primitive wave of hematopoiesis is followed by the definitive wave of hematopoiesis, wherein HSCs give rise to distinct types of blood cells. HSCs can both self-renew and differentiate into all mature blood cell types over the lifetime of an individual [109]. Definitive HSCs arise from hemogenic endothelium, a special population of endothelial cells within the ventral wall of the dorsal aorta that transdifferentiates into HSCs [88]. Specified HSCs budding out from the hemogenic endothelium circulate through the vascular system, eventually homing to the caudal hematopoietic tissue (CHT) where HSCs expand and mature [110]. Afterwards, HSCs migrate to the kidney marrow to act as multipotent blood progenitor cells [111].

To label this HSC population, the Zon group generated three valuable HSC-specific transgenic reporter lines. First, the *Tg(-6.0itga2b:EGFP)* line was originally created to assess zebrafish thrombocytes and prothrombocytes, but turned out to be more specific for the HSC population [86]. The transgenic construct was generated using EGFP driven by the promoter of platelet glycoprotein IIb (*itga2b*) cloned from PAC 166I10. The second transgenic line *TgPAC(myb:2xmyb-EGFP)* was generated by homologous recombination of a 3.7-kb EGFP construct into the UTR precisely before the start site of a PAC clone containing *cmyb* [112]. Combination of *TgPAC(myb:2xmyb-EGFP)* and endothelium-specific *Tg(lmo2:DsRed)* labels HSCs at 36 hpf [112]. Finally, the Zon group generated *Tg(Mmu.Runx1:EGFP)* and *Tg(Mmu.Runx1:NLS-mCherry)*, wherein the mouse Runx1 + 23 enhancer drives either EGFP or nuclear mCherry. The double transgenic line *Tg(Mmu.Runx1:EGFP); Tg(Mmu.Runx1:NLS-mCherry)* showed consistent expression of both fluorescent proteins, confirming the HSC specific expression of the reporter in the lines [110]*.* To generate the transgenic construct, the Runx1 + 23 enhancer was amplified from C57/BL6 mouse genomic DNA [113]. Subsequently, the resulting Runx1 + 23 enhancer DNA fragment and the β-globin minimal promoter were simultaneously cloned into the Tol2 plasmid. These HSC-specific transgenic lines have been used to study embryonic HSC emergence [88, 114–116], thrombocyte development [117], environmental regulation of HSCs [110], mechanosensory regulation of HSC specification [118, 119], brain development related to DNA damage [120] and leukemia [121].

In addition to the aforementioned reporter lines transiently expressing markers of HSC populations, several transgenic lines that permanently label HSC precursors have been also generated to understand the origin of HSCs with lineage-tracing studies. To test whether HSCs arise from endothelial cells in the zebrafish embryo, the Traver group used a permanent marking system consisting of a floxed reporter transgene and a Cre line driven by the endothelium-specific *kdrl* upstream promoter/enhancer elements [88]. The transgenic construct was generated by cloning a 6.8-kb DNA fragment of the *kdrl* promoter/ enhancer sequences [122] into the upstream region of a Cre recombinase. The resulting construct *Tg(kdrl:Cre)* was subcloned into meganuclease plasmid vector, which was then linearized and injected into one-cell-stage zebrafish embryos. In parallel, *Tg(actb2:LOXP-STOP-LOXP-DsRedEx)*, a Cre-mediated genetic switch line, was generated with a 10.5-kb upstream fragment of the β-actin promoter/enhancer followed by a 5.7-kb floxed STOP cassette and DsRed [88]. DsRed serves as a reporter for Cre-mediated removal of the STOP cassette. The transgenic construct was ligated into the Tol2 vector [123] and the final construct was co-injected with Tol2 mRNA into one-cell stage embryos to produce the transgenic founders. This switch line undergoes excision of the STOP cassette in *Cre-*expressing cells, permanently marking these cells and their progeny with DsRed expression. Using these combinatorial transgenic lines, Bertrand et al. demonstrated that HSCs originate from embryonic endothelium in the adult kidney marrow. All types of blood cells from zebrafish whole kidney marrow expressed DsRed, suggesting that all the blood lineages arise from endothelial population. In addition, these lines have also been used in endocardial lineage tracing during zebrafish heart regeneration [124].

Furthermore, the Traver group narrowed down the source of HSC populations in the hemogenic endothelium in the ventral wall of the dorsal aorta. Using *gata2b* as the key marker for this HSC precursor cells, they generated *TgBAC(gata2b:KALTA4)* to trace cells arising from the hemogenic endothelium [125]. The Gal4 variant KalTA4 was inserted before the start site of *gata2b* on the BAC through BAC recombineering [126, 127], and the modified BAC plasmid was injected with Tol2 mRNA into *Tg(4xUAS:GFP)* embryos. Subsequently, the candidate founders were screened for GFP expression in the endothelium. To permanently label *gata2b*-specific hemogenic endothelium for lineage tracing, Butko et al. used triple transgenic zebrafish lines *TgBAC(gata2b:KALTA4); Tg(UAS:Cre, cryaa:Venus); Tg(actb2:LOXP-STOP-LOXP-DsRedEx)*. In this triple transgenic line, tissue-specifically expressed GAL4 turns on the UAS promoter to induce Cre, which in turn activates permanent DsRed switch in the hemogenic endothelium. The presence of DsRed^+^ cells in the CHT, thymus and kidney in 3 dpf embryos indicates that HSCs originate from the *gata2b*-specific hemogenic endothelium in the dorsal aorta [125].

#### Lymphoid transgenic lines

Unlike the primitive wave of hematopoiesis, lymphopoiesis originates solely from definitive HSC progenitor cells [128]. *rag1* and *rag2* are traditionally used markers for lymphocyte populations during early zebrafish development and are expressed in the thymus and adult kidney where zebrafish lymphocyte maturation occurs [129, 130]. The Lin group generated lymphoid lineage specific transgenic reporter lines using these *rag1* and *rag2* drivers [131, 132]. First, *Tg(rag1:GFP)* was generated using reporter constructs containing a 4.7-kb fragment 5´ of the zebrafish *rag1* TSS. Chi-induced homologous recombination [133] inserted the GFP gene into the PAC containing the *rag1* promoter [131]. In addition, to generate *Tg(rag2:GFP)* construct*,* a 6.5-kb fragment of the *rag2* TSS was subcloned from the PAC into the 5′region of a modified GFP reporter gene (GM2) on pBluescript KS(-) plasmid [132]. These lymphoid-specific lines have been used to study lymphocyte development [86], olfactory neuron development [132] and T cell leukemia [134, 135].

#### Summary

Hematopoiesis is highly conserved in zebrafish and mammals. Over the past two decades, diverse blood-specific transgenic reporter lines along with advanced imaging technologies, FACS sorting analysis and various genetic approaches have helped further our understanding of the development and physiological roles of hematopoietic cell lineages. The increasing number of more tailored transgenic fluorescent zebrafish lines is anticipated to widen our understanding of hematopoiesis ontogeny.

### The nervous system

#### Habenula transgenic lines

The habenula is an evolutionarily conserved brain area of the limbic system that connects the telencephalic nuclei to the brain stem nuclei, such as the interpeduncular nucleus (IPN), ventral tegmental area, and raphe [136]. It is implicated in various behaviors such as pain, stress, anxiety, sleep and reward [137]. Furthermore, the habenulae of many vertebrates show left–right asymmetry with respect to size and neuronal circuits, making it a good model to examine brain asymmetry [138]. To study the physiological roles and asymmetrical development of the habenula, transgenic animal models in which the habenula can be labeled and manipulated are required.

To verify the asymmetrical innervations of the zebrafish dorsal habenula, a homologous region to the mammalian medial habenula [139], Okamoto et al. cloned a 5-kb 5′ upstream sequence and a 0.3-kb intron of the *pou4fl* gene that encodes a POU-domain transcription factor into the upstream region of the zebrafish *hsp70* promoter and EGFP sequences [140], resulting in creation of the *Tg(pou4f1-hsp70l:GFP)* construct (Table 4). Subsequently, the linearized construct was microinjected into zebrafish embryos to produce transgenic zebrafish [141], in which the medial subdomain and the axonal projections of the dorsal habenula were labeled [140, 142, 143]. The labeled medial subdomain was larger in the right dorsal habenula than in the left [140]. Moreover, the axonal projections from the GFP-expressing neurons specifically terminated in the ventral and intermediate IPN [140].

**Table 4:** Transgenic reporter zebrafish lines targeting the nervous system

Construct name	Construct ID	Transgenic name	Transgenic ID	Target	References
*Tg(pou4f1-hsp70l:GFP)*	ZDB-TGCONSTRCT-070117-170	*Tg(pou4f1-hsp70l:GFP)*^*rw0110b*^	ZDB-ALT-050317-18	Medial division of the dorsal habenula	[140]
*TgBAC(aoc1:GAL4-VP16)*	ZDB-TGCONSTRCT-150312-5	*TgBAC(aoc1:GAL4-VP16)*^*rw0148a*^	ZDB-ALT-150312-5	Ventral habenula	[145]
*Tg(olig2:EGFP)*	ZDB-TGCONSTRCT-070117-167	*Tg(olig2:EGFP)*^*vu12*^	ZDB-ALT-041129-8	OLs, RG, MNs	[150]
*Tg(elavl3:EGFP) / Tg(HuC:EGFP)*	ZDB-TGCONSTRCT-070117-150	*Tg(elavl3:EGFP) / Tg(HuC:EGFP)*^*knu3*^	ZDB-ALT-060301-2	Differentiated neurons (motor and sensory)	[140]
*Tg(elavl3:Kaede) / Tg(HuC:Kaede)*	ZDB-TGCONSTRCT-070117-93	*Tg(elavl3:Kaede) / Tg(HuC:Kaede)*^*rw0130a*^	ZDB-ALT-060619-4	Differentiated neurons (motor and sensory	[160]
*Tg2(elavl3:GAL4) / Tg2(HuC:GAL4)*	ZDB-TGCONSTRCT-130919-1	*Tg2(elavl3:GAL4) / Tg2(HuC:GAL4)*^*zf409*^	ZDB-ALT-130919-2	Differentiated neurons (motor and sensory	[161]
*Tg(elavl3:GCaMP3) / Tg(HuC:GCaMP3)*	ZDB-TGCONSTRCT-131122-1	*Tg(elavl3:GCaMP3) / Tg(HuC:GCaMP3)*^*ens100*^	ZDB-ALT-131122-1	Differentiated neurons (motor and sensory	[162]
*Tg(1mbp:EGFP)*	ZDB-TGCONSTRCT-120103-2	*Tg(1mbp:EGFP)*^*ue1*^	ZDB-ALT-120103-1	Myelin sheaths, OLs and Schwann cells	[163]
*Tg(mbp:EGFP-CAAX)*	ZDB-TGCONSTRCT-120103-3	*Tg(mbp:EGFP-CAAX)*^*ue2*^	ZDB-ALT-120103-2	Myelin sheaths, OLs and Schwann cells	[167]
*Tg(mbpa:GAL4-VP16)*	ZDB-TGCONSTRCT-150803-1	*Tg(mbpa:GAL4-VP16)*^*km5*^	ZDB-ALT-150803-1	Myelin sheaths, OLs and Schwann cells	[168]
*Tg(UAS:Eco.NfsB-mCherry)*	ZDB-TGCONSTRCT-110215-5	*Tg(UAS:Eco.NfsB-mCherry)*^*rw0144*^	ZDB-ALT-110215-7	Gal4/UAS-NTR system	[168]
*Tg(-4.9sox10:EGFP)*	ZDB-TGCONSTRCT-070117-69	*Tg(-4.9sox10:EGFP)*^*ba2*^	ZDB-ALT-050913-4	Neural crest derived cells (Schwann, DRG) and OL lineage cells	[180]
*Tg(gfap:GFP)*	ZDB-TGCONSTRCT-070117-154	*Tg(gfap:GFP)*^*mi2001*^	ZDB-ALT-060623-4	RGs in brain, SC, retina and peripheral spinal nerves	[182]
*Tg(gfap:NTR-mCherry)*	ZDB-TGCONSTRCT-160630-2	*Tg(gfap:NTR-mCherry)*^*scz059*^	ZDB-ALT-160630-2	RGs in brain, SC, retina and peripheral spinal nerves	[186]
*Tg(gfap:Cre-ERT2,cryaa:YFP)*	ZDB-TGCONSTRCT-150902-1	*Tg(gfap:Cre-ERT2,cryaa:YFP)*^*zd16*^	ZDB-ALT-150902-1	RGs in brain, SC, retina and peripheral spinal nerves	[187]
*Tg(-3.9nes:GFP) / Tg(-3.9nestin:GFP)*	ZDB-TGCONSTRCT-100308-3	*Tg(-3.9nes:GFP) / Tg(-3.9nestin:GFP)*^*zf168*^	ZDB-ALT-100308-4	NSCs	[183]
*Tg(gfap:EGFP)*	ZDB-TGCONSTRCT-070410-2	*Tg(gfap:EGFP)*^*nt11*^	ZDB-ALT-070410-12	RGs in brain, SC, retina and peripheral spinal nerves	[183]
*TgBAC(nes:EGFP)*	ZDB-TGCONSTRCT-110309-7	*TgBAC(nes:EGFP)*^*tud100*^	ZDB-ALT-110309-7	NSCs	[191]
*Tg(UAS-E1B:DsRed,Hsa.MAPT_P301L)*	ZDB-TGCONSTRCT-111028-1	*Tg(UAS-E1B:DsRed,Hsa.MAPT_P301L)*^*mde3*^	ZDB-ALT-111028–1	Tauopathic neurons (CNS neurons)	[195–199]
*Tg(slc6a3:EGFP)*	ZDB-TGCONSTRCT-111206-9	*Tg(dat:EGFP) / Tg(slc6a3:EGFP)*^*ot80*^	ZDB-ALT-111206-10	Dopaminergic neurons	[202]
*Tg(slc6a3:CFP-NTR)*	ZDB-TGCONSTRCT-160128-1	*Tg(dat:CFP-NTR) / Tg(slc6a3:CFP-NTR)*^*ot1413*^	ZDB-ALT-160128-7	Dopaminergic neurons	[204]

In addition, Okamoto et al. developed a BAC construct to label and manipulate the zebrafish ventral habenula, a homologous region to the mammalian lateral habenula [139]. The GAL4-VP16 sequence was introduced via BAC recombineering into the BAC clone (CH211-172G6) [144] containing the zebrafish *amine oxidase copper containing 1 (aoc1)* gene, a ventral habenula specific gene [139], resulting in *TgBAC(aoc1:GAL4-VP16)* construct [145]. To facilitate its genomic integration, the BAC construct was modified further by introducing an iTol2 cassette via homologous recombination, which was then used to generate transgenic fish [146]. GAL4-VP16 driven GFP^+^ cells were specifically localized in the ventral habenula, and their axons projected to the median raphe [145]. In this transgenic fish, optogenetics [147] and targeted toxin expression [145] can label or functionally deactivate the ventral habenula.

#### Oligodendrocyte transgenic lines

Multipotent neural progenitor cells produce diverse kinds of neural cells including interneurons, motor neurons and glial cells during vertebrate development. The progenitor cells of the pMN domain in the spinal cord, a part of the CNS, are the representative cell clusters for studying mechanisms underlying early neuroglia development. Since this population is specified by an oligodendrocyte lineage transcription factor 2 (Olig2), Shin et al. generated an *olig2* reporter zebrafish line with *Tg(olig2:EGFP)* construct that was created by inserting EGFP into a BAC clone harboring *olig2* regulatory sequences (NCBI accession number NM_178100) via BAC recombineering [148, 149]. This line visualizes progenitor cells and radial glia (RG) of the pMN domain, motor neurons (MNs), oligodendrocyte lineage cells in the spinal cord at early developmental stages, and oligodendrocytes (OLs) in the adult stage [150]. It has been extensively used to study the developmental mechanisms of MNs and OLs from *olig2*^+^ progenitor cells [148, 151], regeneration of MNs after injury [152, 153] and the specification and differentiation of OLs [154]. In addition, this transgenic line has been exploited to mark cerebrospinal fluid contacting neurons in the spinal cord [155] and eurydendroid cells in the cerebellum [156].

#### HuC transgenic lines

HuC, a homolog of *Drosophila elav*, is a vertebrate neuron-specific gene [157] that plays important roles in post-mitotic differentiation and maintenance of neuronal cells [158]. As HuC is critical during the early stage of neuronal differentiation, its reporter line was needed to study zebrafish neurogenesis. Thus, Park et al. cloned a 4.6-kb genomic DNA fragment upstream of zebrafish *HuC* into the 5′ end of the start codon of the GFP gene in CS2A(-) plasmid. The resulting construct *Tg(huc:gfp)* was linearized and microinjected into zebrafish embryos to generate the transgenic line [159], in which all differentiated neurons in the CNS, including motor and sensory neurons, were labeled [159]. This transgenic line was used to determine the developmental status of neurons [159]. The Huc promoter derived from *Tg(huc:gfp)* construct has been extensively used to develop diverse transgenic lines such as *Tg(huc:kaede)* using photoactivatable fluorescent proteins for lineage tracing [160], *Tg(huc:gal4)* using GAL4/UAS transactivation system for the targeted gene expression [161], and *Tg(huc:GCamp3)* for the detection of neuronal activity [162].

#### Myelination transgenic lines

Myelin sheaths are lipid-rich, multilayered membranes around axons, and are generated by OLs in the CNS and Schwann cells in the peripheral nervous system (PNS). They are required for nerve conduction velocity, protection of the axons from environmental risk factors, and supply of some energy sources such as lactate. Upon acute injury, myelin sheaths are lost, yet later on they are regenerated by myelinating glial cells. However, in chronic demyelinating conditions such as multiple sclerosis (MS), regeneration of myelin sheaths is practically impossible. Therefore, treatment of demyelinating diseases entails our deeper understanding of formation of the myelin sheaths. Jung et al. generated transgenic zebrafish to visualize myelinating glial cells and myelin sheaths in the zebrafish CNS and PNS [163]. They constructed *Tg(mbp:egfp)* by cloning a 2-kb regulatory element of the zebrafish *myelin basic protein (mbp)* into a Tol2-GFP vector lacking the cytomegalovirus (CMV) promoter [163] and established a transgenic line using transposon-mediated integration [164]. In this line, as expected, GFP specifically labeled myelin sheaths and OLs and Schwann cells from embryos to adulthood [163]. This transgenic model has aided in the investigation of the development of OLs in vivo [165] and been used for high-resolution screening of chemical compounds that regulate myelination [166]. In addition, the *mbp* promoter in *Tg(mbp:egfp)* was used to develop a *Tg(mbp:EGFP-CAAX)* line using membrane-targeted EGFP to study the dynamics of myelination [167]. Furthermore, using a chemogenetic ablation system, demyelination zebrafish models such as *Tg(mbp:gal4vp16;uas:nfsb-mcherry)* have been generated [168, 169].

#### Neural crest cell (NCC) transgenic lines

NCCs originate from the neural plate border and migrate to diverse locations to form various cell lineages such as neurons and glia in the PNS, craniofacial cartilage, and smooth muscle [170]. SRY-box transcription factor 10 (Sox10) is a key transcription factor for studying the ontogeny of NC [171] as Sox10 mutations are associated with developmental defects in PNS neurons, ganglia, myelin sheath, and melanocytes [172–174]. Carney et al. generated a transgenic line to monitor NC-derived cells in vivo using the *Tg(sox10:egfp)* construct [175]. They cloned a 4.9-kb genomic fragment of the zebrafish *sox10* promoter sequences into the XLT.GFPLT.CS2 + vector containing the *egfp* gene, and then generated a transgenic line using the linearized construct. The resulting line marked *sox10*^+^ NC-derived cells, including Schwann cells, dorsal root ganglion and cranial ganglia [175], uncovered a role of Sox10 in the development of sensory neurons [175], and revealed mechanism underlying NC migration during craniofacial morphogenesis [176, 177]. As Sox10 is necessary for the differentiation of OLs in the CNS [178], this transgenic line also labels OL lineage cells [179] and has been used to identify novel genes regulating the development of OL with a microarray-based assay [179].

#### RG transgenic lines

RG are an early form of glial cells observed during the vertebrate neural development, give rise to intermediate neural progenitors and neurons, such as neural stem cells (NSCs), and act as scaffold cells to help the new neurons migrate towards their target locations in the CNS [180, 181]. Thus, defects in the development and function of RG cause neurodevelopmental diseases such as lissencephaly (which means “smooth brain”) [181], highlighting that RG is critical to CNS development. Bernardos et al. generated transgenic zebrafish, in which GFP expression was driven by the regulatory sequences of zebrafish *glial fibrillary acidic protein (gfap)*. They cloned *gfap* regulatory sequences into the pEGFP-1 vector (Clontech), which was linearized and then microinjected into zebrafish embryos. The resulting *Tg(gfap:GFP)* line displays RG in the brain, spinal cord, retina and the peripheral spinal nerves [182]. In addition, this line has been used to study the characterization of neural progenitor cells in the adult brain [183], the behavior of NSCs in the normal and injured adult brain [184], and functions of Müller glia in the adult retina [185]. Finally, the *gfap* promoter has been used to generate several other transgenic lines, such as *Tg(gfap:nfsb-mcherry)* that can ablate RG [186] and *Tg(gfap:Cre*^*ERT2*^*)* that can track RG lineages [187].

#### NSC transgenic lines

NSCs play a crucial role in the production of various types of neurons and glia for the formation of the nervous systems and the maintenance of the stem cell niche, which is a key regulator of stem cells in neurogenesis [188]. The mechanisms underlying generation of various differentiated cell types in the CNS have garnered interest. Nestin, an intermediate filament protein, is a widely employed marker of multipotent NSCs [189]. Lam et al. established a transgenic line expressing GFP under the control of the *nestin* promoter [183]. They cloned a 3.9-kb *nestin* regulatory region into the pCS2:GFP-*Sce*I vector, and the resulting *Tg(nestin:gfp)* construct was microinjected with I-*Sce*I meganuclease [50, 183]. The *Tg(nestin:gfp)* line labeled the progenitor cells with RG-like morphology and self-renewal capacity located in the ventricular zone of the adult CNS [183]. This line has been also used to study the heterogeneity of neural progenitor cells in the adult brain [190] and the regeneration of the injured brain and spinal cord in adult zebrafish [103, 190]. Finally, Kaslin et al. developed a *TgBAC(nestin:GFP)* zebrafish line using BAC recombineering to investigate the stem cell niche in adult cerebellum [191].

#### Tau transgenic lines

Tau is a microtubule-associated protein, is abundantly expressed in the CNS neurons, and maintain the stability of microtubules in neuronal axons [192]. These proteins undergo biochemical modifications such as phosphorylation for cytoskeletal plasticity [193]. However, abnormally phosphorylated tau proteins form neurofibrillary tangles that lead to cell death and thus several neurodegenerative diseases, including Alzheimer's disease and chronic traumatic encephalopathy [194]. To develop therapeutic agents to treat and prevent disease progression, it is important to investigate the underlying mechanism of their pathologies. Paquet et al. generated transgenic zebrafish that recapitulate the pathological features of tauopathy [195]. To generate the tauopathy model, they first employed a bidirectional expression system based on the Gal4/UAS system, which allows for simultaneous expression of transgenes in forward or reverse orientations [195]. They inserted the *E1b* promoter into the flanking sites of the UAS promoter and then cloned human TAU-P301L and DsRed fluorescent protein gene in both directions of the UAS promoter in the pT2KXIGdeltaIN plasmid, resulting in construction of *Tg(UAS-E1B:DsRed,Hsa.MAPT_P301L)* plasmid, with which the cognate transgenic line was established using Tol2-mediated integration. This line was used to study a role for BDNF signaling in tauopathy [196], microglial dynamics against tauopathic neurons [197], and the mechanism by which pathological tau proteins aggregate [198, 199].

#### Dopaminergic neuron transgenic lines

Dopaminergic neurons, which synthesize dopamine (DA) from the amino acid tyrosine, play important roles in the regulation of several physiological and behavioral processes, including voluntary movement, mood, reward, addiction, and memory in the CNS [200]. Deficiency of DA and loss of dopaminergic neurons in the substantia nigra elicit several neurological disorders such as Parkinson’s disease (PD) and impaired motor activity [201]. Therefore, it is necessary to study the pathological mechanisms of PD in association with the development of DA neurons. To label DA neurons in vivo, Xi et al. cloned EGFP gene into *dopamine transporter (dat)* exon 1 in the PAC clone containing the 13-kb of regulatory sequences of *dat* and transferred the resulting PAC-EGFP to pGEM-Tol2 vector [202], thereby generating Tol2-PAC-EGFP construct, with which they created the *Tg(dat:EGFP)* line using Tol2-mediated transgenesis. This line expresses EGFP under the control of *dat* regulatory elements and thus labeled DA neurons in the ventral diencephalon, retina, olfactory bulb, pretectum and caudal hypothalamus [202]. In this line, individual DAT-expressing neurons can be tracked before and after treatment with L-DOPA, a precursor of dopamine [203]. Moreover, a *Tg(dat:CFP-NTR)* line that can ablate DA neurons chemogenetically was developed to investigate PD with locomotor phenotype [204].

### The urogenital system

#### Introduction

Nephron segment patterning and cellular composition are conserved between zebrafish and mammals [205]. Zebrafish embryos develop functional pronephros by 2 dpf and a fully functioning mature pronephric kidney by 4 dpf [206, 207]. Many lines labeling pronephric structures have been established (Table 5), allowing the precise determination of the location, function, and expression profiles of particular cells in the kidney and providing extraordinary tools to model and visualize the biological processes underlying kidney development and disease [208].

**Table 5:** Transgenic reporter zebrafish lines targeting the urogenital system

Construct name	Construct ID	Transgenic name	Transgenic ID	Target	References
*Tg(wt1b:EGFP)*	ZDB-TGCONSTRCT-071127-1	*Tg(-26wt1b:EGFP)*^*li1*^	ZDB-ALT-071127-1	Pronephric kidney	[215–220]
*Tg(lhx1a:EGFP)*	ZDB-TGCONSTRCT-100323-1	*Tg(lhx1a:EGFP)*^*pt303*^	ZDB-ALT-100323-3	Marginal cells, shield, notochord and bilateral mesoderm	[220, 222, 223]
*Tg2(cdh17:EGFP)*	ZDB-TGCONSTRCT-140116-2	*Tg2(cdh17:EGFP)*^*pt305*^	ZDB-ALT-140116-11	Whole length pronephric tubule	[220]
*Tg(sox10:EGFP)*	ZDB-TGCONSTRCT-150414-3	*Tg(PT:EGFP)*^*nz4*^	ZDB-ALT-150414-3	Proximal pronephric tubule	[225, 226]
*Tg(enpep:EGFP)*	ZDB-TGCONSTRCT-101123-2	*Tg(enpep:GFP)*^*p152*^	ZDB-ALT-101123-3	Pronephric tubules, ducts and podocyte-like cells of glomeruli	[227–231]
*Tg(gtshβ:GFP)*	ZDB-TGCONSTRCT-141007-2	*Tg(Eco.Tshb:EGFP)*^*ihb50*^	ZDB-ALT-141007-2	Proximal pronephric tubule, pituitary gland and adult pronephric duct epithelium	[232, 233]
*Tg(-2.5nphs2:EGFP)*	ZDB-TGCONSTRCT-120613-1	*Tg(podocin:GFP) / Tg(-2.5nphs2:EGFP)*^*ki1*^	ZDB-ALT-120613-1	Podocytes and glomerular epithelial cells	[234–239]
*Tg(nphs2:GAL4-VP16)*	ZDB-TGCONSTRCT-150504-7	*Tg(podocin:Gal4VP16 / Tg(nphs2:GAL4-VP16)*^*fb202*^	ZDB-ALT-150504-5	Podocytes and glomerular epithelial cells	[210, 240, 241]
*Tg(ddx4:ddx4-EGFP)*	ZDB-TGCONSTRCT-070814-1	*Tg(vasa:vasa-EGFP)*^*zf45*^	ZDB-ALT-070814-1	PGCs	[243–245, 252–254]
*Tg(piwil1:EGFP)*	ZDB-TGCONSTRCT-110126-5	*Tg(ziwi:EGFP) / Tg(piwil1:EGFP)*^*uc1*^	ZDB-ALT-110126-6	PGCs	[245, 257–260]
*Tg(piwil1:EGFP-UTRnanos3)*	ZDB-TGCONSTRCT-181226-1	*Tg(piwil1:EGFP-UTRnanos3)*^*ihb327*^	ZDB-ALT-181226-1	PGCs	[261–263]
*Tg(gsdf:EGFP)*	ZDB-TGCONSTRCT-130910-1	*Tg(gsdf:EGFP)*^*inr2*^	ZDB-ALT-130910-2	Sertoli and granulosa cells	[264, 265]

While fish generate nephrons throughout their lifespan and regenerate nephrons de novo after injury, mammals can only partly repair their nephrons and cannot form new ones [209, 210]. As such, fish are an appropriate model to study molecular mechanism underlying renal regeneration, which in turn may lead to development of therapeutic activation of mammalian renal regeneration. Zebrafish renal disease models can be generated with genetic alteration, transient gene knockdown or genome-editing technology. Crossing these zebrafish models to transgenic fluorescent zebrafish lines may yield embryos with abnormal pronephros that can be imaged with in vivo fluorescence microscopy. Zebrafish kidney injury models successfully recapitulate the features of mammalian acute kidney injury (AKI), such as characteristic histological changes, reduced renal function and pericardial edema [211–213].

Development of germline requires the specification and survival of primordial germ cells (PGCs) in the embryo as well as the maintenance of gamete production during the reproductive life cycle of the adult. This process is fundamental to all metazoans, and some components of the genetic pathway regulating germ cell development and function are evolutionarily conserved. Study on this process entails establishment of in vivo methods for functional analysis of genes involved in zebrafish gonad development and creation of transgenic lines to visualize the gonads throughout the zebrafish life cycle.

#### Kidney transgenic lines

The Wilms’ tumor suppressor gene *wt1* is essential for kidney development and highly conserved among vertebrates. However, most fish species possess two *wt1* paralogs, *wt1a* and *wt1b*, while mammals have only one *wt1* gene [214]. Human *WT1* mutations cause Wilms’ tumor, which is childhood kidney cancer, and the developmental anomalies of the urogenital tract. Transgenic lines echoing *wt1* gene expressions profile have been generated to examine the structure and development of the zebrafish embryonic kidney, the pronephros, and the roles of the *wt1* genes in the pronephros development. Among them, the *Tg(wt1b:eGFP)* line was produced by co-injecting into one-cell stage zebrafish embryos I-SceI meganuclease and *Tg(wt1b:eGFP)* plasmid that drives EGFP expression under a 25.9-kb fragment encompassing the entire region between *wt1b* and the 5′ neighboring gene *ga17* minus translational start site of *wt1b* [215]. In this line, GFP expression recapitulates the expression pattern of endogenous *wt1b* in the glomeruli. Also labeled in this line are the pronephric tubules and the proximal regions of the ducts, in which endogenous *wt1b* gene is not observed, allowing for visualization of the entire pronephric kidney encompassing glomeruli, tubules and ducts [216]. Fluorescence imaging of *Tg(wt1b:eGFP)* zebrafish larvae revealed the segmental organization of each nephron into glomerulus, neck, proximal convoluted and straight tubules, corpuscle of Stannius and pronephric duct, which fuse to the cloaca at 2 dpf [217, 218]. The *Tg(wt1b:eGFP)* line has contributed to the research on the development and regeneration of kidney as well as the drug screening against nephrotoxicity and genetic kidney diseases including hereditary glomerulopathies and cystic kidney diseases [217, 219, 220].

The Lim1 homeobox protein, Lhx1, is essential for establishing the kidney field in embryonic development and in kidney regeneration after injury [221]. To develop a quantitative high-content screening assay for agents that increase the Lhx1a expression during development, the *Tg(lhx1a:EGFP)* line was generated through I-SceI meganuclease-mediated transgenesis [222]. This line labels marginal cells, shield, notochord and bilateral intermediate mesoderms that give rise to the kidney, in addition to the polster and the diencephalon. At later stages, this line also marks the pronephric proximal and distal tubules and duct as well as the anterior lateral line ganglia, forebrain, hindbrain, midbrain, otic vesicle, posterior lateral line ganglia, spinal cord and tailbud [222]. The renal progenitors isolated from *T*g(*lhx1a:EGFP*) can generate new nephrons when introduced into a damaged host kidneys [223]. A high-content assay using *Tg(lhx1a:EGFP*) zebrafish embryos was developed employing cognition network technology, an artificial intelligence-based image analysis, that can identify small molecules expanding the kidney field [220]. HDAC inhibitors that facilitate recovery from AKI in zebrafish larvae were screened using this zebrafish system as a regeneration model for drug discovery [220].

The zebrafish homolog of mammalian kidney-specific cadherin, *cdh17*, is expressed in the epithelium and ducts of the entire tubule during larval development and adult [224]. To establish *cdh17* reporter zebrafish, a 5-kb genomic region containing the promoter and the 5′ UTR sequences of the *cdh17* locus was cloned into the 5′ region of the pI-SceI:EGFP plasmid, which was in turn used to generate the *Tg(cdh17:egfp)* transgenic line via I-SceI meganuclease-mediated transgenesis [220]. This line at 48 hpf labels the kidney including a pair of tubular structures that converge at the cloaca and the tubular subdomains, excluding the glomeruli. At later points of development, however, the entire embryonic tubular segments are labelled. Easily detected in this line is kidney field expansion, which helps identify novel agents that may have the potential to augment kidney regeneration after injury [220].

To visualize pronephric tubular injury caused by gentamicin in zebrafish larvae, the *Tg(PT:EGFP)* line was generated by expressing EGFP under the *sox10* promoter [225]. This line labels cells in the proximal pronephric tubule and NCCs [225]. In contrast, the *Tg(cdh17:EGFP*) line labels the whole length of the pronephric tubule [220]. These two lines were combined to identify the spatial expression of molecular markers in the infection-associated AKI [226].

The gene *enpep* encodes glutamyl aminopeptidase implicated in the regulation of blood pressure, blood vessel formation and tumorigenesis. The *Tg(enpep:egfp)* line was generated, in which EGFP expression is driven by the 2.3-kb *enpep* promoter [227]. *Tg(enpep:GFP)* larvae label both pronephric tubules and ducts as well as podocyte-like cells of the glomeruli in early development [227]. This line has been used to identify and characterize the genes associated with pronephric development, kidney diseases and cellular dynamics during adult zebrafish kidney regeneration [228–231].

To understand a role for *gtshβ* in kidney tubule morphogenesis, the *Tg(gtshβ:GFP)* line was created, in which GFP is expressed under the control of the *gtshβ* promoter [232]. In this line, GFP is expressed in proximal pronephric tubules as well as in pituitary gland during embryogenesis and kidney duct epithelium in adult fish, conferring a tool to study the tubular development with genetic or chemical approaches [232]. This line has deepened our understanding of the signaling pathways that trigger renal tubular damage during lethal lipopolysaccharide (LPS)-induced septic shock in an AKI model, suggesting that this transgenic zebrafish is an ideal model to carry out in vivo screen for potential antisepsis therapeutic strategies [233].

The *Tg(podocin:GFP*) line was generated using the *Tg(-2.5nphs2:EGFP)* construct via Tol2 transposon-mediated transgenesis. EGFP expression is driven by the 2.5-kb zebrafish *podocin* promoter [234]*.* The loss of glomerular GFP expression in the *Tg(podocin:GFP*) embryos indicates developmental aberrations of and injury to the glomerulus [235–239]. To investigate the podocyte injury, several techniques to induce zebrafish AKI are available. For example, *Tg(nphs2:GAL4-VP16)* [240] and *Tg(pod:NTR-mCherry)* [210, 241] lines were established to temporally ablate or injure podocytes, the visceral glomerular epithelial cells, by expressing NTR under the control of the zebrafish *nphs2* promoter. To visualize the spatiotemporal pattern of mesonephrogenesis and investigate the development and postinjury regeneration of the mesonephros in adult zebrafish, cytotoxin was induced in podocytes of larval or adult transgenic zebrafish that expressed NTR under the control of the podocin promoter, eliciting glomerular injury including podocyte loss, reduced expression of podocyte marker genes and foot process effacement [210, 240, 241].

#### Germline and gonadal transgenic lines

*ddx4* [DEAD (Asp-Glu-Ala-Asp) box polypeptide 4], which was previously known as *vasa* or *vas*, is a conserved gene belonging to the DEAD box helicase family. Being specifically expressed in the germline across metazoans, it has been extensively used as a germ cell marker [242]. The *Tg(vasa:vasa-EGFP)* zebrafish was generated using the *Tg(vasa:vasa-eGFP)* or *Tg(ddx4:ddx4-EGFP)* construct that includes the *vasa* regulatory region fused to *egfp* [243]. This line expresses EGFP in the PGCs from 24 hpf onwards [243]. During early development, however, only maternal GFP signal is detected in the germline, and zygotic transcription of *vasa-egfp* driven by the *vasa* regulatory region begins after sexual differentiation when the germ cells enter meiosis [244, 245]. This line has been widely used to investigate roles of the genes involved in gonad development [246–251] as well as the gonadal stem cell features [252, 253]. To visualize the germline in real time and in vivo from 1 dpf through 12 weeks post-fertilization, the *Tg(vasa:vasa-EGFP)* line was combined with zebrafish mutants *mitfa*^w2/w2^ (melanocyte inducing transcription factor a; ZFIN ID ZDB-ALT-990423-22) or *mpv17*^*b18/b18*^ (mitochondrial inner membrane protein 17; ZDB-GENO-141218-8), which renders both larvae and adult zebrafish transparent [254].

*ziwi* encodes an RNA-binding zinc finger protein and similar to *vasa,* it is specifically expressed in the zebrafish germline throughout development [255, 256]. *Tg(piwil1:EGFP)* was generated using the *Tg(piwil1:EGFP)* construct comprising the promoter elements of *ziwi (piwil1)*, the zebrafish homolog for *Drosophila piwi*, fused to *egfp* [245]. In this line, *ziwi:EGFP* is maternally supplied in embryos and zygotic EGFP is first detected around at 7 dpf [245], implying that this line is less dependent on maternal EGFP to label germ cells early in development than the *Tg(vasa:vasa-EGFP)* line. The *Tg(piwil1:EGFP)* line has characterized the roles of the genes involved in gonad development [257–260].

Another transgenic line that can display the PGCs, *Tg(piwil1:egfp-UTRnanos3)*, was generated using the *Tg(piwil1:EGFP-UTRnanos3)* construct [261]. This line labels PGCs at the shield stage, oogonia and oocytes at early stages in the ovary, and spermatogonia, spermatocyte and spermatid at early stage in the testis, thus illuminating the zebrafish germline throughout the lifespan [261]. This line has been utilized to analyze gene functions in gonadal development [262, 263].

*gsdf* is expressed in the Sertoli and granulosa cells. To illuminate these cells, the *Tg(gsdf:EGFP)* line was generated using the *Tg(gsdf:EGFP)* construct including the 2-kb proximal promoter region of *gsdf* via Tol2 transposase-mediated transgenesis [264]. EGFP expression is first detected at 16 dpf and progresses from the posteroventral region lining the swim bladder towards the anteroventral region and the urogenital papilla at 19–42 dpf prior to the sexual differentiation of the gonad [264]. This line has been also employed to visualize the 3D architecture of the testis and its cellular content [265].

### Digestive system

#### Introduction

The zebrafish digestive system can be divided into the gastrointestinal (GI) tract including esophagus, intestinal bulb, mid intestine, posterior intestine and anus, and accessory organs such as liver and pancreas. To investigate developmental process of the digestive system in zebrafish, transgenic fluorescent zebrafish lines have been widely used. Here, we summarize the representative zebrafish transgenic reporter lines that label various cell types in the GI tract, liver and pancreas (Table 6).

**Table 6:** Transgenic reporter zebrafish lines targeting the digestive system

Construct name	Construct ID	Transgenic name	Transgenic ID	Target	References
*Tg(fabp2:RFP)*	ZDB-TGCONSTRCT-110208-2	*Tg(fabp2:RFP)as200*	ZDB-ALT-110208-3	Intestinal bulb and mid intestine	[269, 271–274]
*TgBAC(cldn15la-GFP)*	ZDB-TGCONSTRCT-140613-1	*TgBAC(cldn15la-GFP)*^*pd1034*^	ZDB-ALT-140613-1	Epithelial cells (intestinal bulb to posterior intestine)	[275–279]
*Tg(-8.3bphox2b:Kaede)*	ZDB-TGCONSTRCT-150305-1	*Tg(-8.3bphox2b:Kaede)*^*em2*^	ZDB-ALT-150305-1	ENS	[280–284]
*Tg(-2.8fabp10a:EGFP))*	ZDB-TGCONSTRCT-070117-123	*Tg(-2.8fabp10a:EGFP))*^*zf235*^	ZDB-ALT-110523-10	Hepatocytes	[297–302]
*Tg(EPV.TP1-Mmu.Hbb:EGFP)*	ZDB-TGCONSTRCT-090625-1	*Tg(EPV.TP1-Mmu.Hbb:EGFP)*^*um14*^	ZDB-ALT-090625-1	Intrahepatic biliary cells	[304–308]
*TgBAC(hand2:EGFP)*	ZDB-TGCONSTRCT-110128-8	*TgBAC(hand2:EGFP)*^*pd24*^	ZDB-ALT-110128-40	Hepatic stellate cells	[309–312, 325]
*Tg(ela3l:EGFP)*	ZDB-TGCONSTRCT-070117-92	*Tg(ela3l:EGFP)*^*gz2*^	ZDB-ALT-060710-10	Pancreatic exocrine cells	[313]
*Tg(gcga:GFP)*	ZDB-TGCONSTRCT-070215-2	*Tg(gcga:GFP)*^*ia1*^	ZDB-ALT-070215-2	Pancreatic α-cells	[314, 317, 318]
*Tg(mnx1:GFP)*	ZDB-TGCONSTRCT-070117-40	*Tg(mnx1:GFP)*^*ml2*^	ZDB-ALT-051025-4	Pancreatic β-cells	[315, 316]
*Tg(ins:DsRed)*	ZDB-TGCONSTRCT-080826-1	*Tg(ins:DsRed)*^*m1018*^	ZDB-ALT-080826-1	Pancreatic β-cells	[317, 318]
*Tg(Xla.Eef1a1:GFP)*	ZDB-TGCONSTRCT-070117-34	*Tg(Xla.Eef1a1:GFP)*^*s854*^	ZDB-ALT-030702-2	Digestive tract, liver and pancreas	[298, 320–324]

#### GI tract

The intestinal architecture of the zebrafish closely resembles the mammalian counterpart [266–268]. To understand molecular mechanism underlying development of the intestinal lumen and enteric nervous system (ENS), various transgenic fluorescent zebrafish lines have been generated.

To establish a gut-specific expression of target genes, the Jen-Leih Wu group at Academia Sinica constructed a plasmid harboring 4.5-kb intestine specific promoter of zebrafish intestine fatty acid-binding protein (*I-FABP*) fused to *RFP*. This plasmid was linearized and microinjected into zebrafish embryos to generate the *Tg(fabp2:RFP)* line, which labeled the zebrafish intestinal bulb and mid intestine [269]. His group also reported that the 192-bp region in the *I-FABP* promoter sufficed for gut-specific expression [270]. This *Tg(fabp2:RFP)* line carrying the 4.5-kb promoter has been widely used to study functional organization of zebrafish intestine and human GI diseases such as inflammatory bowel disease and enterocolitis [271–274].

In addition, the Bagnat group at Duke University Medical Center linearized the *TgBAC(cldn15la:GFP)* construct with AsiSI (New England Biolabs) and microinjected the linearized construct into one-cell stage zebrafish embryos to generate the *TgBAC(cldn15la:GFP)* line, which exhibits GFP expression in the membranes of epithelial cells spanning from the intestinal bulb to posterior intestine [275]. This line has been used to uncover the mechanism of the intestinal epithelial formation and patterning as well as intestinal barrier function and inflammation in zebrafish [275–279].

The ENS is the largest part of the vertebrate PNSs. To image the ENS development in real time and in vivo, the Iain Shepherd group at Emory University constructed a plasmid encoding the wavelength-sensitive fluorescent protein Kaede under the control of the enhancer of *paired-like homeobox 2b* (*phox2b*), a gene involved in the development of enteric neuron progenitors [280]. Subsequently, they microinjected the resulting construct with Tol2 transposase mRNA into embryos to generate the *Tg(-8.3bphox2b:Kaede)* line. This line has been used to study de novo enteric neurogenesis in post-embryonic zebrafish, a role of retinoic acid in colonization of the gut by vagal neural crest cells [281, 282], and Hirschsprung disease caused by failure of the ENS [283, 284].

#### Liver

Cell types and metabolic pathways in the liver are comparable between zebrafish and mammals [285–289]. Hence, zebrafish has emerged as an important animal model to study the development [290, 291] and diseases of the liver [292–296]. Transgenic lines that express fluorescent proteins in hepatocytes, intrahepatic biliary cells and hepatic stellate cells have been developed.

To label zebrafish hepatocytes with EGFP, the Wu group at National Taiwan Ocean University cloned the promoter region of *liver-FABP* (*L-FABP*) into the pEGFP-C1. The resulting construct *pLF2.8-EGFP* was used to generate the *Tg(-2.8fabp10a:EGFP)* line [297]. More than 50 studies have taken advantage of this line to investigate liver development. For example, this line was used to determine the role of cellular signaling pathways in liver development [298, 299] as well as drug-induced hepatic injury or genetically-induced hepatic diseases [300–302]. Of note, the Didier Stainier group at University of California, San Francisco established the *Tg(fabp10a:DsRed)* line to study patterning and differentiation of the hepatopancreatic ductal system [303].

To create a Notch reporter line, the Steven Leach group at Johns Hopkins University fused six copies of the *Epstein-Barr Virus terminal protein 1* (*EPV.TP1*) promoter region to *β-globin* minimal promoter and *EGFP* using the Gateway technology. The resulting construct *Tg(EPV.Tp1-Mmu.Hbb:EGFP)* and *Tol2* transposase mRNA were co-microinjected into zebrafish embryos. As a result, the *Tg(EPV.Tp1-Mmu.Hbb:EGFP)* line was generated, which labels intrahepatic biliary cells [304]. This line has been adopted to reveal molecular mechanisms by which endodermal notch signaling, cannabinoid receptor signaling and inhibitor of DNA binding (Id) protein induce bile duct development and liver diseases [305–308].

To generate a transgenic fluorescent zebrafish line that marks hepatic stellate cells, the Stainier group employed the regulatory region of *heart and neural crest derivatives expressed 2* (*hand2*), which encodes a basic helix-loop-helix transcription factor implicated in organ development, determination of intestine left–right asymmetry and nervous system development. A BAC clone harboring the regulatory region of *hand2* was used to generate the *TgBAC(hand2:EGFP)* line by BAC transgenesis. Because activation of hepatic stellate cells plays a key role in regeneration upon hepatic injury, this line has been employed to study liver diseases, to visualize activation of hepatic stellate cells upon ethanol-induced hepatic injury, and to create NTR / MTZ induced hepatic fibrosis models [309–312].

#### Pancreas

The pancreas consists of endocrine and exocrine systems. To study differentiation, proliferation and morphogenesis of exocrine cells, the Gong group at National University of Singapore used the *elastaseA* (*elaA*) regulatory sequence (− 1.8 kb) for exocrine specific expression of GFP. The *Tg(ElaA:EGFP)* plasmid was linearized and microinjected into one-cell stage embryos to generate the *Tg(ela3l:EGFP)* line [313]. The pancreatic endocrine system, pancreatic islets, consists of α-, β- and δ-cells that secrete glucagon, insulin and somatostatin, respectively. The Argenton group at University of Padua used the *glucagon a* (*gcga*) promoter region to generate the *Tg(gcga:GFP)* line that expresses GFP in glucagon producing pancreatic α-cells [314]. Insulin producing pancreatic β-cells can be visualized by the *Tg(mnx1:GFP)* or *Tg(ins:RFP)* line. *mnx1* encodes a homeobox transcription factor that is involved in motoneuron differentiation and pancreas development. The Sanes group at Washington University in St. Louis isolated the regulatory region of *motor neuron and pancreas homeobox 1* (*mnx1*; also called *hb9*) from a PAC clone. To study neuromuscular synapses, the regulatory region was fused to GFP and then microinjected into embryos to establish the *Tg(mnx1:GFP)* line [315]. Later on, the Dirk Meyer group at University of Innsbruck used the *Tg(mnx1:GFP); Tg(ins:dsRed)* line to show that the pancreatic *mnx1* expression precedes expression of *insulin* (*ins*), and to determine the underlying mechanisms of β-cell fate specification [316]. The *Tg(ins:dsRed)* line was generated by the Driever group at University of Freiburg and first reported by the Stainier group [317]. The *Tg(ins:dsRed)* line and *Tg(gcga:GFP);Tg(ins:dsRed)* line have been used to investigate development and regeneration of pancreas [317, 318] as well as to screen a small molecule library to identify enhancers of β-cell regeneration [319].

#### Endodermal organs

The *Tg(Xla.Eef1a1:GFP)* line (more popularly called the gutGFP line) was generated incidentally by the Herwig Baier group at University of California, San Francisco. Microinjection of linearized plasmid pESG that encodes GFP downstream of the *Xenopus* elongation factor (EF)-1α promoter into zebrafish embryos yielded the line. GFP expression in this line was ubiquitous at early stage, yet was restricted to the endoderm, eye and hatching gland by 22 hpf [320]. The insertion site of the transgene has not been characterized yet. As this line expresses GFP in the developing digestive tract, liver and pancreas, most of early research on these organs had benefited from the gutGFP line. Using this line, the Stainier group published three papers on zebrafish liver, pancreas and intestinal epithelium morphogenesis, respectively [320–322]. These papers laid the foundation for the research on the zebrafish GI tract and its accessory organs. As such, it is not surprising that this line has significantly contributed to uncover molecular mechanisms underlying development of these organs. For example, Wnt signaling was shown to be required for liver development and regeneration [298], pancreas associated transcription factor 1a (Ptf1a) was reported to regulate endocrine versus exocrine fate in pancreas development [323], and the target of rapamycin (TOR) signaling was demonstrated to regulate intestinal epithelium morphogenesis [324].

### Intracellular organelles

#### Introduction

Zebrafish is an excellent animal model to study embryogenesis and human diseases because it allows for visualization and manipulation of cellular organelles and processes [326]. Development of organisms is governed by the spatial and temporal regulations of proliferation, apoptosis, migration, differentiation and morphological specialization. Proteins are synthesized at the endoplasmic reticulum, glycosylated at the Golgi complex, and trafficked to target locations, either intracellular or extracellular [327]. Secretory proteins go through the secretory pathway comprising diverse protein complexes at each step to recruit Rab GTPases and SNARE proteins to tether vesicles to target organelles and facilitate membrane fusion [328]. The understanding of this pathway from the perspective of intracellular organelles is fundamental to study the behavior and biology of cells in organisms. Many human genetic diseases such as mitochondriopathies [329] and diseases of protein trafficking [330] are linked to abnormal organelle functions. Hence, development of transgenic reporter lines labeling organelles was required.

#### Nucleus transgenic lines

To visualize overall zebrafish embryonic development, the *Tg(actb2:H2B-GFP)* line was generated (Table 7). Under the regulation of *β-actin* promoter, this line expresses fusion proteins of human histone-2 and GFP that localize to chromatin, and has been used to analyze cell cycle length, nuclear architecture and the temporal dynamics of the nuclear compartment [331]. When driven by tissue specific promoters such as *islet* [*Tg(isl1a:NLS-GFP)*], *mylpfa* [*Tg(mylpfa:Hsa.HIST1H2BJ-GFP*] and *runx* [*(Tg(Mmu.Runx1:NLS-mCherry)*], these lines can easily monitor neuronal, muscular and hematopoietic development in real time and in vivo [332–334].

**Table 7:** Transgenic reporter zebrafish lines targeting intracellular organelles

Construct name	Construct ID	Transgenic name	Transgenic ID	Target	References
*Tg(actb2:H2B-GFP)*	ZDB-TGCONSTRCT-170619-1	*Tg(actb2:H2B-GFP)*^*zf712*^	ZDB-ALT-170619-1	Chromatin (Nucleus)	[331]
*Tg(isl1a:NLS-GFP)*	ZDB-TGCONSTRCT-201203-1	*Tg(isl1a:NLS-GFP)*^*fh558*^	ZDB-ALT-201203-5	Neurons	[334]
*Tg(Mmu.Runx1:NLS-mCherry)*	ZDB-TGCONSTRCT-150512-2	*Tg(Mmu.Runx1:NLS-mCherry)*^*cz2010*^	ZDB-ALT-150512-2	Hematopoietic cells	[110]
*Tg(mylpfa:Hsa.HIST1H2BJ-GFP)*	ZDB-TGCONSTRCT-160728-1	*Tg(mylpfa:Hsa.HIST1H2BJ-GFP)*^*sq32*^	ZDB-ALT-160728-1	Muscular cells	[333]
*Tg(actb2:Hsa.B4GALT1-GFP)*	ZDB-TGCONSTRCT-120419-3	*Tg(actb2:Hsa.B4GALT1-GFP)*^*mss3*^	ZDB-ALT-120419-5	Golgi apparatus	[336]
*Tg(h2afx:EGFP-rab5c)*	ZDB-TGCONSTRCT-111017-3	*Tg(h2ax:EGFP-rab5c)*^*mw5*^	ZDB-ALT-111017-5	Early endosome	[341]
*Tg(h2afx:EGFP-rab7a)*	ZDB-TGCONSTRCT-111017-4	*Tg(h2ax:EGFP-rab7a)*^*mw7*^	ZDB-ALT-111017-6	Late endosome	[341]
*Tg(h2afx:EGFP-rab11a)*	ZDB-TGCONSTRCT-111017-5	*Tg(h2ax:EGFP-rab11a)*^*mw6*^	ZDB-ALT-111017-7	Recycling endosome	[341]
*Tg(CMV:EGFP-map1lc3b)*	ZDB-TGCONSTRCT-091029-1	*GFP-Lc3, Tg(CMV:EGFP-map1lc3b)*^*zf155*^	ZDB-ALT-091029-2	Lysosomes	[349]
*Tg(CMV:EGFP-gabarapa)*	ZDB-TGCONSTRCT-091029-2	*GFP-Gabarap, Tg(CMV:EGFP-gabarap)*^*zf156*^	ZDB-ALT-091029-1	Lysosomes	[349]
*Tg(fabp10a:EGFP-map1lc3b)*	ZDB-TGCONSTRCT-120710-1	*Tg(fabp10a:EGFP-map1lc3b)*^*gz22*^	ZDB-ALT-120710-1	Liver lysosomes	[351]
*Tg(Xla.Eef1α1:GFP-LC3-RFP-LC3ΔG)*	ZDB-TGCONSTRCT-171018-1	*Tg(Xla.Eef1α1:GFP-LC3-RFP-LC3ΔG)*^*jt2*^	ZDB-ALT-171018-4	Lysosomes	[352–354]
*Tg(gnat2:GFP-map1lc3b)*	ZDB-TGCONSTRCT-140416-5	*Tg(gnat2:GFP-map1lc3b)*^*w138*^	ZDB-ALT-140416-5	Photoreceptor cell lysosomes	[337]
*Tg(Xla.Eef1a1:mlsEGFP)*	ZDB-TGCONSTRCT-090309-1	*Tg(Xla.Eef1a1:mlsEGFP)*^*cms1*^	ZDB-ALT-090309-2	Mitochondria	[357]

#### Golgi apparatus transgenic lines

To image the trans-Golgi secretory pathway in the developing embryos in real time and in vivo, the Gerhart group cloned amino acids 1–6 from human B4GALT1 fused to GFP to the region downstream of the zebrafish semi-ubiquitous *β-actin* promoter using Gateway technology, thereby creating *Tg(β-act:GalT-GFP)*. Galactotransferase (GalT) is a Golgi-retained enzyme. Using this construct and Tol2 mediated transgenesis [335], they generated the *Tg(actb2:Hsa.B4GALT1-GFP)* line [336], which has been exploited to study dynamic protein secretory pathways that influence cell proliferation during ontogeny, and regeneration [337, 338].

#### Endosome transgenic lines

Rab proteins mediate endocytosis and vesicle trafficking in development, disease and cellular homeostasis [339]. More than 65 Rab genes have been identified to date and are essential communicators with their effector proteins in formation, motilities and tethering of vesicles in membrane-bounded organelles [340]. To facilitate imaging of endosomes in real time and in vivo, Clark et al. generated transgenic fluorescent zebrafish lines [341]. To label early, recycling and late endosomes, they chose *Rab5c* (early), *Rab11a* (recycling), and *Rab7* (late) based on localization and functions of these endosome subtypes in zebrafish and other species.

RAB5 has been studied for receptor-mediated signaling pathway in vesicle transport and fusion of membrane between clathrin coated vesicles of the plasma membrane and newly formed endosomes [342]. Among five zebrafish paralogs (*rab5a-c, rab5aa* and *rab5ab*) of *Drosophila Rab5*, *rab5c* was selected for an early endosomal marker due to its ubiquitous expression in zebrafish embryos [343]. RAB11 protein is localized to the pericentriolar-recycling compartment, the recycling endosome [342]. Zebrafish *rab11* has four paralogs: *rab11a*, *rab11a-like, rab11ba* and *rab11bb*. Of which, Rab11a was used as a recycling endosome marker due to its highest protein sequence similarity to *Drosophila* Rab11a. In addition, Rab7 is localized to late endosomes and implicated in biogenesis of late endosomes and targeting trafficking vesicles to degradation [342]. Unlike Rab5c and Rab11a, the zebrafish Rab7 has no paralog. Rab5c, Rab11a and Rab7 were N-terminally fused to EGFP individually and then cloned downstream of the quasi-ubiquitous h2afx (also known as h2ax) promoter [341]. The resulting constructs were used to generate respective transgenic fluorescent zebrafish lines via Tol2 mediated transgenesis [335]: *Tg(h2afx:EGFP-Rab5c)*, *Tg(h2afx:EGFP-rab11a)* and *Tg(h2afx:EGFP-Rab7)*. These lines were validated with co-localization between the fluorescent puncta from the EGFP-Rab lines and lipophilic dye FM4-64 at the early, recycling and late endosomes, respectively. Furthermore, these lines have been utilized to study the endocytosis, recycling endosomes of protein secretory pathway and their intracellular dynamics in development [344] and human diseases [231].

#### Autophagy transgenic line

Autophagy is an essential cellular degradative pathway that delivers cytoplasmic cargo to the lysosome. During autophagy, an autophagosome, a double-membrane vesicle, fuses with a lysosome, hydrolyses, and then degrades the macromolecules, which in turn recycles back into the cytosol for use as cellular nutrients. Autophagy plays an important role in host immune defense, tumor suppression, cardiovascular disease, gastrointestinal disorders, neurodegeneration and longevity [345–348]. The real-time monitoring of autophagy is an essential tool to reveal the process of autophagosome formation and to determine clearance of aggregate-prone proteins in in vivo small molecule screens for the autophagy regulators. To this end, He et al. generated two transgenic fluorescent zebrafish lines expressing a GFP fused to Lc3 [*Tg(CMV:EGFP-map1lc3b)*] or to Gabarap N-terminally [*Tg(CMV:EGFP-gabarapa)*] under the control of the constitutive CMV promoter [349]. Gabarap is gamma-aminobutyric acid type A receptor-associated protein. When they treated 3 dpf *Tg(cmv:GFP-Lc3)* embryos with rapamycin and lysosomal inhibitors, they observed that GFP puncta were formed and translocated to lysosomes. These lines have been adopted to monitor autophagic activity during embryogenesis in real time and in vivo [348, 350]. In addition, expression of *GFP-Lc3* in a tissue specific manner [liver: *Tg(fabp10:EGFP-Lc3)*; photoreceptor: *Tg(TαCP:GFP-Lc3)*] has made it feasible to study tissue-specific autophagy [337, 351].

To measure autophagic activity in real time and in vivo, the Mizushima group generated the* Tg(Xla.Eef1α1:GFP-LC3-RFP-LC3ΔG)* construct. Once translated, this gene product is cleaved by ATG4 protease into GFP-LC3 and RFP-LC3ΔG. The former is conjugated with phosphatidylethanolamine (PE), targeted to the autophagosomes, and then degraded upon fusion of the autophagosomes with the lysosomes. However, RFP-LC3ΔG cannot be conjugated with PE due to lack of C-terminal glycine, which renders RFP-LC3ΔG stable in the cytoplasm. As such, the GFP/RFP signal ratio can act as readout of autophagic activity. Using this construct, they generated a *Tg(Xla.Eef1α1:GFP-LC3-RFP-LC3ΔG)* line via Tol2 mediated transgenesis [352]. This line has been employed to assess effect of 1-phenyl 2-thiourea (PTU) on autophagic flux in zebrafish embryos [353] and to demonstrate requirement of autophagy for maturation of surfactant-containing lamellar bodies in the zebrafish swim bladder [354].

#### Mitochondria transgenic line

Mitochondria are double-membrane bound organelles and play an important role in energy production, metabolism, apoptosis, calcium storage and cellular signaling [355]. As such, mitochondria research has been a hot field and thus a need arose to image mitochondria in zebrafish. The Choi group cloned the mitochondrial localization sequence (MLS) of zebrafish *cytochrome c oxidase subunit VIIIA* (*cox8a*) (NCBI accession number NM_001303053.1) to the 5′ end of EGFP gene on a mini-Tol2 vector [356, 357]. Its expression was driven by a *Xenopus* EF-1 α promoter, a ubiquitous promoter. The resulting construct *Tg(Xla.Eef1α1:mlsEGFP)* was used to generate a zebrafish line expressing mitochondrially targeted GFP via Tol2 transposon-mediated transgenesis [164]. As expected, mitochondria in almost all types of cells in this line were labeled with GFP and mitochondrial fragmentation in chemically induced apoptosis was able to be imaged in real time and in vivo [357]. This line has been employed to visualize mitochondria in motor neurons [358], erythroid cells [359], photoreceptor cells [360] and skeletal muscles [361], and to study degradation of mitochondrial GFP [362] and effect of polycyclic aromatic hydrocarbon and hypoxia on mitochondrial content [363]. In addition, MLS-EGFP derived from the construct *Tg(Xla.Eef1α1:mlsEGFP)* was used to image dopaminergic neurons [364, 365], Mauthner cells [366], mitophagy [367] and sensory neurons [368].

## Conclusion and perspectives

This review summarized the transgenic fluorescent zebrafish lines that have been broadly used in biomedical research. As our understanding of promoters and enhancers widens, CRISPR/Cas9 mediated knock-in technology makes translational fusion easier than ever, and super resolution microscopes become commercially available, transgenic fluorescent zebrafish would make it feasible to image and track each and every protein in real time and in vivo in the near future. In addition, spatiotemporal regulation of fluorescent protein expression could be achieved by combining the transgenic fluorescent zebrafish lines with chemical or physical induction of fluorescent protein expression, which may become an invaluable tool in biomedical research.

## Data Availability

Not applicable.

## Abbreviations

AKI: Acute kidney injury
AP: Anterior posterior
BAC: Bacterial artificial chromosome
BMP: Bone morphogenetic protein
BRE: BMP response element
Cas9: CRISPR-associated protein 9
CFP: Cyan fluorescent protein
CHT: Caudal hematopoietic tissue
CMV: Cytomegalovirus
CNC: Cranial neural crest
CNS: Central nervous system
COVID-19: Coronavirus disease-19
CRISPR: Clustered regularly interspaced short palindromic repeats
DA: Dopamine
dat: Dopamine transporter
dn-fgfr1: Dominant negative Fgf receptor
DNA: Deoxyribonucleic acid
dpf: Days post fertilization
DsRed: Discosoma sp. red
*E. coli*: *Escherichia coli*
EF-1 α: Elongation factor-1α
EGFP: Enhanced green fluorescent protein
ENS: Enteric nervous system
EPV.TP1: Epstein-Barr virus terminal protein 1
ER: Endoplasmic reticulum
FACS: Fluorescence-activated cell sorting
FGF: Fibroblast growth factor
FOXP2: Forkhead box protein P2
GalT: Galactotransferase
GFAP: Glial fibrillary acidic protein
GFP: Green fluorescent protein
GI: Gastrointestinal
HDAC: Histone deacetylase
hpf: Hours post fertilization
HSC: Hematopoietic stem cells
HSP: Heat shock protein
I-FABP: Intestine fatty acid-binding protein
ICM: Intermediate cell mass
IPN: Interpeduncular nucleus
LEF: Lymphoid enhancer-binding factor
LFABP: Liver fatty acid binding protein
LPS: Lipopolysaccharide
MBP: Myelin basic protein
MH: Midbrain-hindbrain
MLS: Mitochondrial localization sequence
MN: Motor neurons
MPEG: Macrophage Expressed Gene 1
MPX: Myeloperoxidase
MS: Multiple Sclerosis
MTZ: Metronidazole
NC: Neural crest
NCBI: National Center for Biotechnology Information
NLS: Nuclear localization sequence or signal
NSC: Neural stem cell
NTR: Nitroreductase
OL: Oligodendrocyte
PAC: P1-derived artificial chromosome
PD: Parkinson’s disease
PGC: Primordial germ cell
PLM: Posterior lateral mesoderm
PNS: Peripheral nervous system
PTU: 1-Phenyl 2-thiourea
RBC: Red blood cell
RG: Radial glia
RNA: Ribonucleic acid
RUNX2: Runt-related transcription factor 2
SARS-CoV-2: Severe acute respiratory syndrome coronavirus 2
SOX9: SRY-Box transcription factor 9
TCF: T cell factor proteins
*Tg*: Transgenic
TGF-β: Transforming growth factor beta
TOR: Target of rapamycin
TSS: Transcription start site
UAS: Upstream Activation Sequence
WISH: Whole mount in situ hybridization
ZFIN: Zebrafish information network
